# TPM1 drives cytoskeleton-immunometabolism coupling and LGALS9/CD45-mediated neuroinflammatory propagation in retinitis pigmentosa

**DOI:** 10.1126/sciadv.aea6467

**Published:** 2026-05-27

**Authors:** Rong Li, Jun-Qi Fan, Bin Lin

**Affiliations:** ^1^ School of Optometry, The Hong Kong Polytechnic University, Hong Kong, China.; ^2^ Research Centre for SHARP Vision (RCSV), The Hong Kong Polytechnic University, Hong Kong, China.

## Abstract

Retinitis pigmentosa (RP), the most prevalent inherited retinal degeneration, features progressive photoreceptor loss with no approved disease-modifying therapies. While microglia-driven neuroinflammation accelerates RP progression, its sustaining mechanisms remain elusive. Through integrated multiomics profiling of retinal degeneration 10 (rd10) mice, we identify tropomyosin 1 (TPM1) as a previously unrecognized cytoskeletal-immune regulator orchestrating spatial neuroinflammation in RP. Genetic ablation of *Tpm1* attenuated microglial reactivity and preserved vision, whereas overexpression triggered self-reinforcing inflammation via four interlocked axes: (i) TPM1-mediated activator protein-1 (AP-1) hyperactivation initiates senescence-associated secretory phenotype (SASP) through mitogen-activated protein kinase (MAPK) kinase/extracellular signal–regulated kinase 3–dependent MAPK signaling; (ii) SASP subsequently mediates reduced phagocytosis; (iii) *Tpm1-Apoe/Fabp5* axis disruption precipitates lipid droplet accumulation with cholesterol crystallization; (iv) galectin-9 (LGALS9)/CD45-mediated intermicroglial signaling propagates inflammatory signals across the retina. Our work redefines TPM1 as a linchpin in self-sustaining neurodegeneration cycles, where cytoskeletal dysfunction fuels immunometabolic collapse. These findings unveil precision therapeutic strategies targeting TPM1 hubs—notably the LGALS9/CD45 axis—to disrupt inflammatory cycles while preserving retinal homeostasis.

## Teaser

TPM1 drives RP neurodegeneration via cytoskeletal-immune networks, and targeting TPM1/LGALS9-CD45 preserves vision.

## INTRODUCTION

Retinitis pigmentosa (RP), a heterogeneous group of inherited retinal dystrophies affecting 1 in 4000 individuals, is characterized by progressive and irreversible photoreceptor degeneration (*1*, *2*), ultimately leading to blindness. While mutations in more than 60 genes have been identified in RP (*2*, *3*), the convergent pathways driving nonautonomous photoreceptor death remain enigmatic. This mechanistic ambiguity persists despite three decades of research, leaving more than 1.5 million patients worldwide without disease-modifying therapies (*4*).

Emerging evidence positions retinal microglia—the central nervous system (CNS)–resident immune cells—as critical amplifiers of photoreceptor degeneration (*5*, *6*). In RP models, microglia undergo rapid morphological transformation, migrating to the outer nuclear layer (ONL) where they exhibit impaired phagocytic capacity, aberrant cytokine secretion [tumor necrosis factor–α↑ (TNF-α↑) and interleukin-1β↑ (IL-1β↑)] (*7*), and complement-mediated synaptic stripping (*8*). Pharmacological suppression of microglia delays photoreceptor loss in retinal degeneration 1 (rd1) and rd10 models by 40 to 70% (*9*, *10*), yet current approaches lack cellular specificity and disrupt homeostatic functions. Crucially, the molecular triggers driving pathogenic microglial polarization in RP remain undefined, representing a critical barrier to developing spatially and temporally precise targeted interventions.

Recent breakthroughs in neurodegenerative disease research implicate actin-binding proteins as key regulators of neurodegenerative pathophysiology (*11*–*16*). Tropomyosin 1 (TPM1), a 284–amino acid protein governing actin filament stability, shows notable dysregulation in Alzheimer’s disease (AD) and Parkinson’s disease (PD) (*13*, *14*). Postmortem AD brains exhibit 5.3-fold higher TPM1 level in the white matter, correlating with dystrophic morphology and impaired Aβ clearance (*11*, *13*). In PD models, TPM1 is involved in regulating neurite outgrowth and microglial immunotoxicity (*14*, *17*). Our recent work revealed age-dependent TPM1 accumulation in aging and AD retinas, where it modulates microglial inflammation and photoreceptor apoptosis (*15*, *16*). However, TPM1’s role in inherited retinal degenerations remains unexplored—a critical knowledge gap given the shared neuroinflammatory hallmarks between RP and CNS neurodegenerative disorders.

Here, through integrated single-cell transcriptomics, spatial proteomics, and functional validation in the rd10 RP model, we resolve TPM1’s dual role as both cytoskeletal organizer and inflammatory master regulator. Our multiomics dissection reveals TPM1-overexpressing microglia as pathogenic subpopulation driving RP progression through spreading inflammatory signals to neighboring cells. We delineate a previously unrecognized TPM1-LGALS9/CD45 signaling axis, which selectively suppresses inflammation while preserving microglial homeostasis. This mechanism-driven precision therapeutics addresses persistent clinical challenges: targeted mitigation of neuroinflammatory cascades without inducing broad immunosuppression, providing a critical insight into controlling microglia-mediated inflammation to resolve chronic neuroinflammation and concurrently sustain microglial homeostasis in patients with RP.

## RESULTS

### Spatiotemporal dysregulation of microglial *Tpm1* drives neuroinflammation in RP

**Fig. 1.**
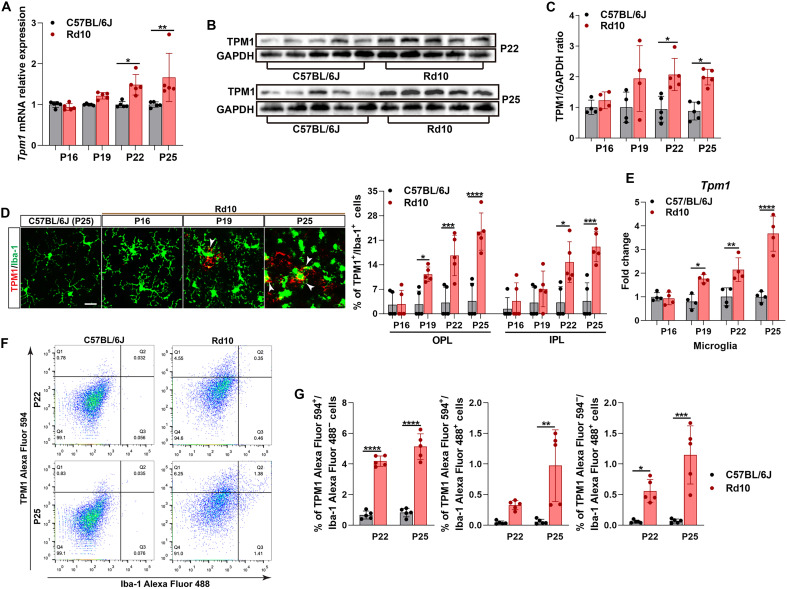
Spatiotemporal dysregulation of microglial *Tpm1* drives neuroinflammation in RP. (**A**) Quantitative polymerase chain reaction (qPCR) analysis of *Tpm1* expression in the retinas of rd10 and age-matched C57BL/6J mice at P16, P19, P22, and P25 (*n* = 5 mice per group). (**B** and **C**) Western blot (B) and quantification (C) of TPM1 expression in the retinas of rd10 and age-matched C57BL/6J mice (*n* = 4 to 5 mice per group). (**D**) Retinal wholemount immunofluorescence showing TPM1^+^ microglia (Ionized calcium-binding adapter molecule 1 (Iba-1)^+^) in both OPL and inner plexiform layer (IPL) of the retinas (*n* = 5 mice per group). The white arrowheads show TPM1^+^ microglia. Four sampling areas with 638.9 μm–by–638.9 μm squares along the dorsal-ventral axes of retinal wholemounts at 200 μm and 1 mm from the optic nerve head on both sides were photographed. Scale bar, 20 μm. (**E**) *Tpm1* expression in fluorescence-activated cell sorting (FACS)–isolated microglia from the retinas of rd10 and age-matched C57BL/6J mice. The results represent four independent experiments. (**F** and **G**) Flow cytometry analysis (F) and quantification of TPM1 Alexa Fluor 594^+^/Iba-1 Alexa Fluor 488^−^, TPM1 Alexa Fluor 594^+^/Iba-1 Alexa Fluor 488^+^, and TPM1 Alexa Fluor 594^−^/Iba-1 Alexa Fluor 488^+^ cells (G) in the retinas of rd10 and age-matched C57BL/6J mice (*n* = 5 mice per group). All data are presented as the means ± SEMs and were analyzed via two-way analysis of variance (ANOVA) with Tukey’s multiple-comparison test (*P < 0.05, **P < 0.01, ***P < 0.001, and ****P < 0.0001).

Emerging evidence implicates microglial-mediated neuroinflammation as a critical driver of retinal degeneration in RP (*7*, *18*). While our prior work identified tropomyosin 1 (*Tpm1*) as a proaging regulator of microglial activation (*15*, *16*), its spatiotemporal dynamics and functional role in RP pathogenesis remain unexplored. Notably, longitudinal analysis revealed progressive TPM1 accumulation in rd10 retinas, with 0.49- to 1.26-fold increases on mRNA (Fig. 1A) and protein levels (Fig. 1, B and C), respectively, at postnatal day 22 (P22)–to–P25 versus age-matched C57BL/6J controls. Spatial mapping uncovered TPM1 amplification specifically within microglia in the outer plexiform layer (OPL)—a critical site of photoreceptor synapse remodeling. Quantification analysis revealed 3.16- to 5.41-fold expansions of TPM1-expressing (TPM1^+^) microglia in the OPL of rd10 retinas from P19 to P25 (Fig. 1D), coinciding with dynamics of photoreceptor loss (*19*, *20*). Single-cell resolution confirmed this dysregulation: Transcriptomic profiling of isolated microglia from rd10 retinas showed sustained *Tpm1* up-regulation from P19 to P25 (Fig. 1E), and flow cytometry revealed 15.88-fold increases in the number of TPM1^+^ microglia in rd10 retinas at P25 (Fig. 1, F and G; *P* < 0.01). Together, these data establish TPM1 as a key regulator of microglia-mediated neuroinflammation and photoreceptor degeneration, implicating its spatiotemporal dysregulation as a pathogenic driver in RP.

### Targeted *Tpm1* knockdown suppresses neuroinflammatory cascades and preserves retinal integrity in RP

**Fig. 2.**
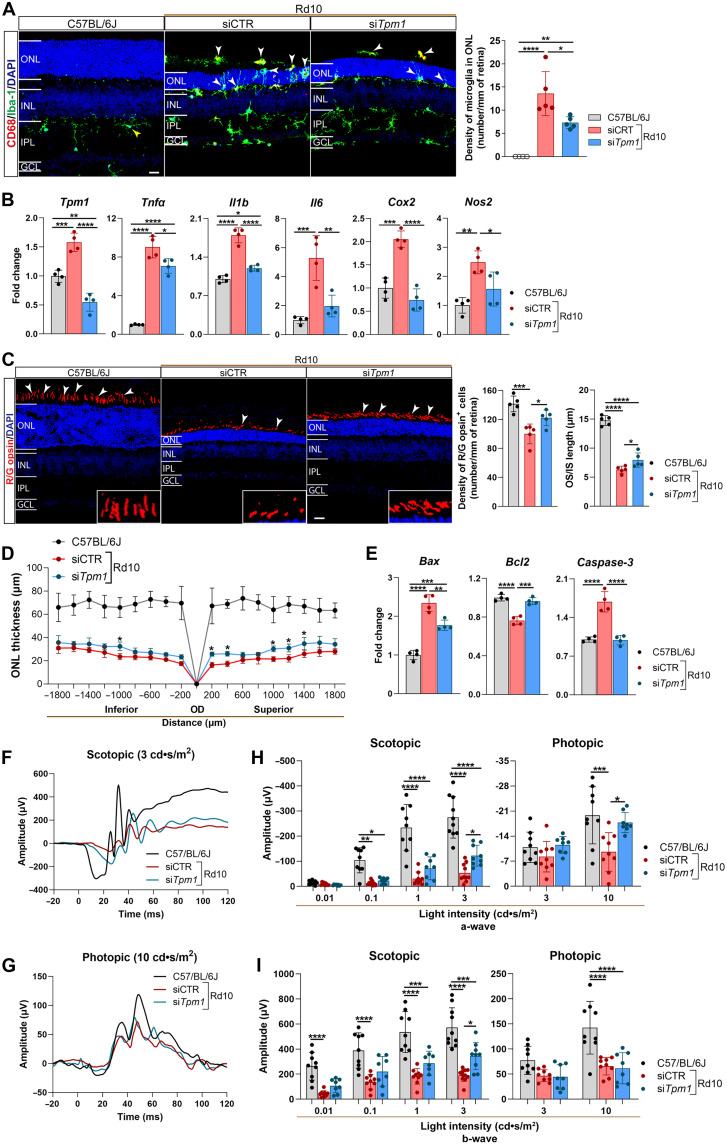
Targeted *Tpm1* knockdown suppresses neuroinflammatory cascades and preserves retinal integrity in RP. (**A**) Retinal sections stained with CD68 and Iba-1 antibodies and quantification of CD68^+^ microglia in the ONL of the retinas from P25 rd10 mice after intravitreal injection of si*Tpm1* or siCTR and from age-matched C57BL/6J mice (*n* = 4 to 5 mice per group). The white arrowheads show CD68^+^ microglia. The intravitreal injections of si*Tpm1* or siCTR were administered to P16 rd10 mice three times at 72-hour intervals over a 9-day treatment period, and the retinas were analyzed at P25. Scale bar, 20 μm. (**B**) qPCR analysis of *Tpm1*, *Tnf*α, *Il1b*, *Il6*, *Cox2*, and *Nos2* expression (*n* = 4 mice per group). (**C**) Retinal sections stained with R/G opsin antibody and quantification of the number of red/green (R/G) opsin^+^ cone cells and the outer/inner segment (OS/IS) length. Boxed areas are magnified below; white arrowheads indicate R/G opsin^+^ cells. Scale bar, 20 μm. (**D**) Spider plots of ONL thickness measured at 200- to 1800-μm intervals from the optic disk (OD) toward superior/inferior retina (*n* = 5 mice per group). (**E**) qPCR analysis of *Bax*, *Bcl2*, and *Caspase-3* expression in the retinas (*n* = 4 mice per group). (**F** to **I**) Representative electroretinogram (ERG) graphs (F and G) and quantification of a- (H) and b-wave (I) amplitudes under both scotopic and photopic conditions on P25 rd10 mice after intravitreal injection with si*Tpm1* or siCTR and age-matched C57BL/6J mice (*n* = 8 to 9 mice per group). For image acquiring (A to C), three views in each retinal section at 100 μm (central), 1 mm (middle), and 1.8 mm (peripheral) from the optic nerve head along the dorsal and ventral directions were captured. The data are presented as the means ± SEMs and were analyzed via one-way [(A) to (C) and (E)] or two-way [(D), (H), and (I)] ANOVA with Tukey’s multiple-comparison test (*P < 0.05, **P < 0.01, ***P < 0.001, and ****P < 0.0001). INL, inner nuclear layer; GCL, ganglion cell layer.

To establish the therapeutic potential of *Tpm1* intervention in RP, we intravitreally delivered *Tpm1*-specific small interfering RNA (siRNA) (si*Tpm1*) into rd10 mouse eyes. *Tpm1* knockdown markedly reduced microglial activation in rd10 retinas, evidenced by decreasing the density of CD68^+^ microglia by 46.0 and 35.1% in the ONL (Fig. 2A) and OPL, respectively, compared to control siRNA (siCTR) controls (fig. S1A). Crucially, it inhibited microglial migration from their native OPL territory to the ONL where photoreceptors are localized (46.0%↓; *P* < 0.05), effectively restoring spatial immune homeostasis (Fig. 2A and fig. S1A). Moreover, *Tpm1* knockdown in rd10 retinas suppressed multiple proinflammatory mediators including cytokines *Tnf*α (21.6%↓), *Il1b* (33.2%↓), and *Il6* (62.7%↓) and enzymes *Cox2* (63.8%↓) and *Nos2* (37.1%↓) versus siCTR controls (Fig. 2B). This broad-spectrum anti-inflammatory effect underscores *Tpm1* as a central regulator of neuroinflammation in RP.

Moreover, targeted-*Tpm1* knockdown conferred multilayered neuroprotection in RP. Specifically, *Tpm1* suppression in rd10 retinas significantly increased cone cell density (22.2%↑; *P* < 0.05) and preserved the length of cone outer/inner segments (OS/IS) (26.3%↑; *P* < 0.05) versus siCTR (Fig. 2C). Meanwhile, it reduced the number of terminal deoxynucleotidyl transferase (TdT)–mediated deoxyuridine triphosphate nick end labeling (TUNEL)^+^ cells in the ONL (38.5%↓) (fig. S1, B and C) and increased ONL thickness (Fig. 2D) and dysregulated apoptosis-associated signature genes (*Bax*, 24.6%↓; *Caspase-3*, 41.2%↓; *Bcl2*, 26.3%↑) (Fig. 2E). *Tpm1* knockdown in rd10 mice markedly restored retinal function, evidenced by increasing a- and b-wave amplitudes (a-wave: 129.6%↑; b-wave: 79.9% ↑) versus siCTR controls under scotopic condition at light intensities of 3 cd•s/m^2^ (Fig. 2, F to I, and fig. S1, D and E).

To dissect the cell-autonomous role of *Tpm1* in microglial dysfunction, we engineered *Tpm1* knockdown in BV2 microglia (30.4% efficiency; fig. S2, A and B), revealing its regulatory mechanism. *Tpm1* suppression reduced lipopolysaccharide (LPS)–induced proinflammatory cascades with notable specificity: *Tnf*α (11.3%↓), *Il1b* (30.4%↓), *Il6* (7.8%↓), and *Cox2* (19.5%↓) versus siCTR controls (fig. S2, A to C). While LPS enhanced microglial motility by 49.3%, *Tpm1* knockdown induced 62.4% inhibition (*P* < 0.01) (fig. S2, D and E), implicating *Tpm1* in cytoskeletal remodeling during immune surveillance. Notably, *Tpm1* depletion increased viable cell numbers by 2.1% (*P* < 0.01) under inflammatory stress (fig. S2F), highlighting context-dependent regulation of survival pathways. These findings position *Tpm1* as a key node coordinating microglial immune reactivity, spatial navigation, and fate determination.

Together, these findings demonstrate that *Tpm1* suppression restores microglial homeostasis, blocks neuroinflammatory cascade, and preserves both structural and functional integrity of photoreceptors in RP.

### *Tpm1* amplification in microglia drives a neuroinflammatory-apoptotic axis in RP

**Fig. 3.**
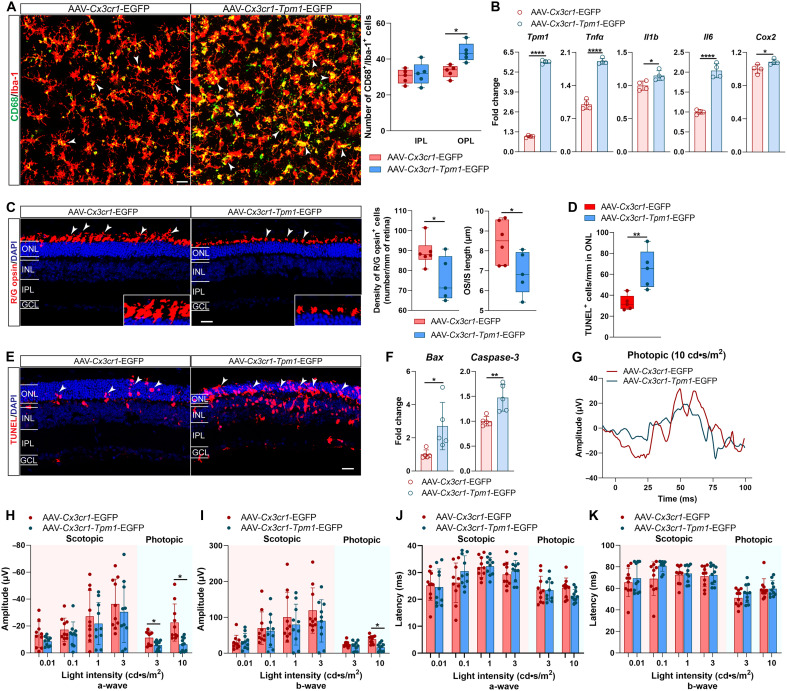
*Tpm1* amplification in microglia drives a neuroinflammatory-apoptotic axis in RP. (**A**) Retinal wholemounts stained with CD68 and Iba-1 antibodies and quantification of CD68^+^ microglia in both OPL and IPL of the retinas from P25 rd10 mice after subretinal injection with AAV-*Cx3cr1-Tpm1*-EGFP or AAV-*Cx3cr1*-EGFP (*n* = 5 mice per group). White arrowheads show CD68^+^ microglia. Four sampling areas with 638.9 μm–by–638.9 μm squares along the dorsal-ventral axes of retinal wholemounts at 200 μm and 1 mm from the optic nerve head on both sides were photographed. (**B**) qPCR analysis of *Tpm1* and proinflammatory cytokines expression (*n* = 4 mice per group). (**C**) Retinal section stained with R/G opsin antibody and quantification of R/G opsin^+^ cells and OS/IS length in the retinas of P25 rd10 mice after subretinal injection with AAV-*Cx3cr1-Tpm1*-EGFP or AAV-*Cx3cr1*-EGFP (*n* = 5 to 6 mice per group). The white arrowheads show R/G opsin^+^ cells. The boxed regions are shown at higher magnification at the bottom. (**D** and **E**) TUNEL staining (E) and quantification of TUNEL^+^ cells in the ONL (D) of the retinas (*n* = 5 mice per group). The white arrowheads show TUNEL^+^ cells. (**F**) qPCR analysis of *Bax* and *Caspase-3* expression in the retinas (*n* = 5 mice per group). (**G** to **K**) The representative ERG graphs (G) and quantification of a- and b-wave amplitudes (H and I) and latencies (J and K) under both scotopic and photopic conditions in P25 rd10 mice after subretinal injection with AAV-*Cx3cr1-Tpm1*-EGFP or AAV-*Cx3cr1*-EGFP (*n* = 10 mice per group). Scale bars, 20 μm (A, C, and E). For image acquiring (C and E), three views in each retinal section at 100 μm (central), 1 mm (middle), and 1.8 mm (peripheral) from the optic nerve head along the dorsal and ventral directions were captured. The data are presented as the means ± SEMs and were analyzed via unpaired two-tailed Student’s *t* tests [(A) to (D) and (F)] and two-way ANOVA with Tukey’s multiple-comparison test [(H) to (K)] (*P < 0.05, **P < 0.01, and ****P < 0.0001).

To establish a causal link between microglial *Tpm1* dysregulation and RP progression, we specifically overexpressed *Tpm1* in microglia of rd10 retinas via subretinal injection with AAV-*Cx3cr1*-*Tpm1*-EGFP (35.5 ± 5.3% transfection efficiency; fig. S3A). We observed that *Tpm1* overexpression resulted in microglial hyperactivation: 31.9% increase of CD68^+^ microglial cell density within the OPL (*P* < 0.05 versus AAV-*Cx3cr1*-EGFP controls; Fig. 3A) and more CD68^+^ microglial cells with hypertrophic soma and shorten dendrites (Fig. 3A). Meanwhile, microglial *Tpm1* overexpression in rd10 retinas triggered proinflammatory cytokine amplification: *Tnf*α (91.7%↑), *Il1b* (15.1%↑), *Il6* (103.6%↑), and *Cox2* (8.5%↑) (*P* < 0.05; Fig. 3B).

Functionally, microglial *Tpm1* overexpression accelerated photoreceptor death in RP. Subretinal injection with AAV-*Cx3cr1-Tpm1*-EGFP in rd10 mice led to apoptosis of cone photoreceptors: 15.2% reduction in cone cell density and 18.3% shortening of cone OS/IS length (*P* < 0.05 versus AAV-*Cx3cr1*-EGFP controls; Fig. 3C). Meanwhile, we observed that microglial *Tpm1* up-regulation in rd10 mice drove apoptosis cascade: 98.2% increase of TUNEL^+^ cell surge in the ONL (*P* < 0.01; Fig. 3, D and E), coactivation of *Bax* (170.5%↑) and *Caspase-3* (47.2%↑) (*P* < 0.05; Fig. 3F), and significant ONL thinning (P < 0.01; fig. S3B). Electrophysiological assessment revealed functional deterioration in rd10 mice following microglial *Tpm1* amplification: decreased photopic a- and b-wave amplitudes at light intensity of 10 cd•s/m^2^ (57.5 to 71.9%↓, *P* < 0.05; Fig. 3, G to K), correlating with photoreceptor cell loss.

This gain-of-function model establishes *Tpm1* as both (i) a critical target for intercepting neuroinflammatory cascades and (ii) a bidirectional regulator of photoreceptor survival, linking microglial hyperactivation to apoptotic degeneration in RP.

### Single-cell transcriptomics reveals *Tpm1*-driven microglial heterogeneity in RP

**Fig. 4.**
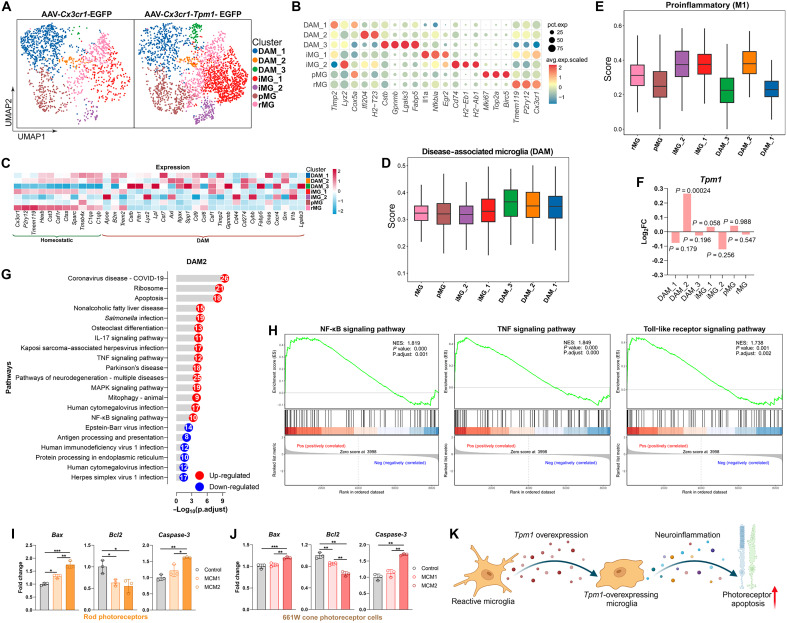
Single-cell transcriptomics reveals *Tpm1*-driven microglial heterogeneity in RP. (**A**) Uniform manifold approximation and projection (UMAP) plot showing seven unique microglial clusters in rd10 mouse retinas at P25 after subretinal injection with AAV-*Cx3cr1-Tpm1*-EGFP or AAV-*Cx3cr1*-EGFP. (**B**) Bubble chart showing marker gene expression in each cluster. (**C**) Heatmap showing expression of homeostatic and DAM genes in different clusters. (**D** and **E**) Box plots showing the UCell score of DAM (D) and M1 proinflammatory (E) pathways in different clusters. (**F**) Quantification of *Tpm1* expression in different microglial clusters between AAV-*Cx3cr1-Tpm1*-EGFP–treated and AAV-*Cx3cr1*-EGFP–treated rd10 retinas. (**G**) KEGG pathway analysis of DEGs in cluster DAM2. (**H**) GSEA analysis of NF-κB, TNF, and Toll-like receptor signaling pathways in cluster DAM2. (**I** and **J**) qPCR analysis of *Bax*, *Bcl2*, and *Caspase-3* expression in rod photoreceptors isolated from P25 C57BL/6J retinas (I) and in 661W cone photoreceptors (J) after treatment with microglial conditioned medium 1 (MCM1) and MCM2. The results shown represent three independent experiments. (**K**) Graphical illustration showing that inflammation in *Tpm1*-overexpressing microglia exacerbates photoreceptor cell death. The data are presented as the means ± SEMs and were analyzed via one-way ANOVA with Tukey’s multiple-comparison test (*P < 0.05, **P < 0.01, and ***P < 0.001).

High-resolution single-cell RNA sequencing (scRNA-seq) profiling of 3757 retinal microglia from rd10 mice following adeno-associated virus (AAV)–mediated *Tpm1* overexpression in microglia unveiled seven functionally distinct clusters (Fig. 4, A and B). The canonical homeostatic makers *Cx3cr1*, *Tmem119*, *P2ry12*, *Trem2*, and *C1qa* were highly and widely expressed among all microglial clusters (fig. S4A). On the basis of the marker gene expression in each cluster, we defined one cluster of resting microglia (rMG) with quiescent metabolic signatures (Fig. 4C and fig. S4B); one cluster of proliferated microglia (pMG) that specifically expressed proliferation marker genes including *Mki67*, *Top2a*, and *Birc5* (Fig. 4B) and showed high score in proliferation pathway (fig. S4B); three disease-associated microglia (DAM) subsets (DAM_1, DAM_2 and DAM_3) that expressed high levels of DAM marker genes including *Timp2*, *Cstb*, *Lgals3*, *Fabp5*, and *Itgax* (Fig. 4, B and C) and presented high score in DAM pathway (Fig. 4D); two clusters of inflammatory-associated microglia (iMG) that highly expressed inflammation-related marker genes including *Il1a*, *Nfkbia*, *Egr2*, *H2-Eb1*, and *H2-Ab1* (Fig. 4B) and showed high score in proinflammatory (M1) pathway (Fig. 4E). In particular, we found that *Tpm1* was significantly up-regulated in DAM2 [log_2_FC (fold change) = 0.27, *P* = 0.00024; AAV-*Cx3cr1-Tpm1*-EGFP versus AAV-*Cx3cr1*-EGFP controls; Fig. 4F], suggesting DAM2 as the *Tpm1* regulatory nexus. Geno Ontology (GO) analysis of differentially expressed genes (DEGs) in DAM2 revealed that up-regulated DEGs were primarily associated with cell apoptosis pathway, cytokines activity, metabolic pathway, and nuclear factor κB (NF-κB) binding, while down-regulated DEGs were related to negative regulation of immune-associated pathway and protein binding (fig. S4C). Kyoto Encyclopedia of Genes and Genomes (KEGG) and gene set enrichment analysis (GSEA) pathway analysis in DAM2 cluster revealed that inflammation- and apoptosis-associated pathways including NF-κB, TNF, Toll-like receptor, and apoptosis- and mitogen-activated protein kinase (MAPK) signaling pathways were significantly up-regulated (Fig. 4, G and H, and fig. S4D), implicating DAM2 as a *Tpm1*-dependent microglial phenotype driving RP progression.

To directly test the role of *Tpm1-*overexpressing microglia in photoreceptor death, we overexpressed *Tpm1* in isolated microglia from 6-week-old C57BL/6J mouse brains by transfection with *Tpm1*-specific plasmid (45.0% transfection efficiency; fig. S4E). We observed that *Tpm1* overexpression triggered inflammatory priming in microglia: *Tnf*α (44.8%↑), *Il1b* (55.4%↑), *Il6* (63.1%↑), and *Cox2* (71.4%↑) versus control plasmid (fig. S4E). Notably, when stimulating isolated rod photoreceptors from P25 C57BL/6J retinas or 661W cone photoreceptor cells with conditional medium collected from microglia treated with *Tpm1* plasmid [microglial-conditioned medium 2 (MCM2)] or control plasmid (MCM1), we found that MCM2 stimulation significantly up-regulated proapoptosis genes *Bax* (15.7 to 35.1%↑; *P* < 0.05) and *Caspase-3* (33.4 to 52.1%↑; *P* < 0.05), down-regulated the anti-apoptosis gene *Bcl2* (22.3%↓; *P* < 0.01), and reduced cell viability (32.6 to 41.7%↓; *P* < 0.05) in both rod and 661W cone photoreceptors compared to MCM1-treated controls (Fig. 4, I and J, and fig. S4F), suggesting that *Tpm1* as a gatekeeper of microglia-photoreceptor cross-talk via proinflammatory cascades.

Together, these results indicate that *Tpm1* up-regulation triggers distinct microglial heterogeneity, with DAM2 emerging as a TPM1-dependent pathogenic subpopulation. Moreover, *Tpm1* orchestrates photoreceptor apoptosis through paracrine inflammatory signaling in RP, positioning it as a gatekeeper of microglia-photoreceptor cross-talk in RP (Fig. 4K).

### *Tpm1* orchestrates microglial senescence and reduced phagocytosis in RP pathogenesis

**Fig. 5.**
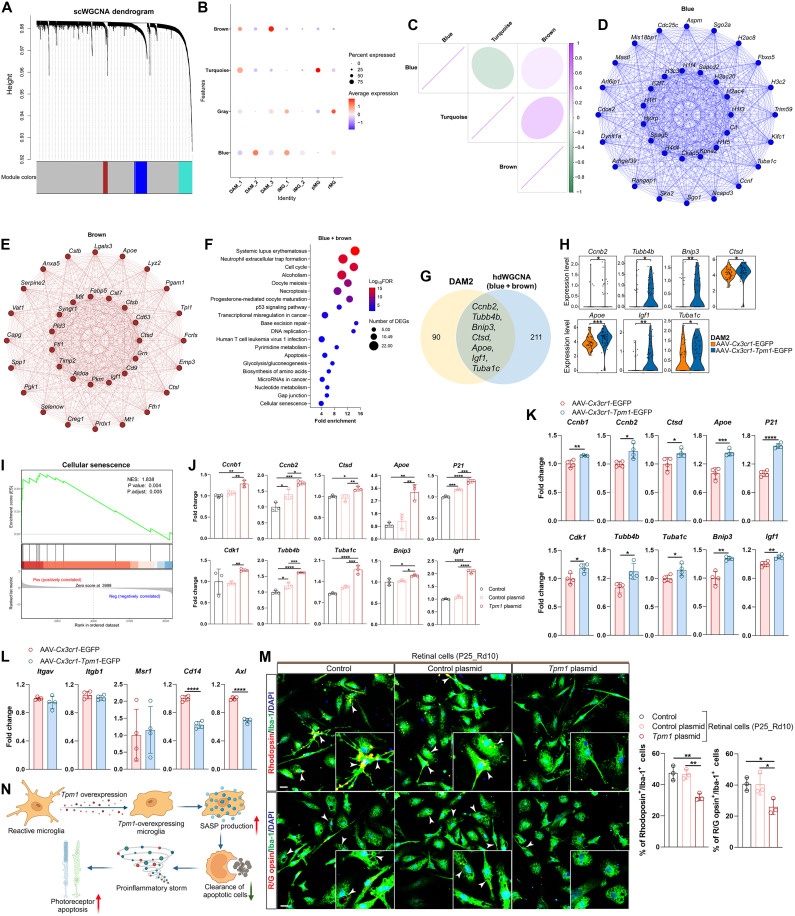
*Tpm1* orchestrates microglial senescence and reduced phagocytosis in RP pathogenesis. (**A**) signed consensus weighted gene coexpression network analysis (scWGCNA) in all *Tpm1*-expressing microglia. All *Tpm1*-expressing microglia were categorized into blue, gray, turquoise, and brown modules. (**B**) Expression of these four modules in different microglial clusters. (**C**) Correlation diagram showing the interrelationship among all coexpression modules. (**D** and **E**) Coexpression plots depicting top 35 genes in module blue (D) and brown (E). (**F**) KEGG pathway analysis of genes in module blue and brown. (**G**) Venn diagram showing coexpressed genes between DAM2 and module blue and brown. (**H**) Violin plots showing expression of senescent genes *Ccnb2*, *Tubb4b*, *Bnip3*, *Ctsd*, *Apoe*, *Igf1*, and *Tuba1c* in cluster DAM2. (**I**) GSEA analysis of cellular senescence in cluster DAM2. (**J**) qPCR analysis of senescent genes in microglia isolated from 6-week-old C57BL/6J mice and transfected with *Tpm1* plasmid or control plasmid. (**K**) qPCR analysis of above senescent genes in the retinas of rd10 mice (*n* = 4 mice per group). (**L**) qPCR analysis of phagocytosis-associated genes in the retinas (*n* = 4 mice per group). (**M**) Immunostaining of primary microglia with rhodopsin or R/G opsin and Iba-1 antibodies and quantification of microglia phagocytizing rod or cone photoreceptors after transfection with *Tpm1* plasmid or control plasmid in primary microglia followed by coculture with retinal cells isolated from P25 rd10 retinas. The white arrowheads indicate the engulfment of rod or cone photoreceptors by microglia. Insets: High-magnification views. Scale bars, 20 μm. The results shown represent three to four independent experiments. The data are presented as the means ± SEMs and were analyzed via unpaired two-tailed Student’s *t* tests [(H), (K), and (L)] or one-way ANOVA with Tukey’s multiple-comparison test (J and M) (*P < 0.05, **P < 0.01, ***P < 0.001, and ****P < 0.0001). (**N**) Graphical illustration depicting that up-regulation of senescence and impairment of microglial phagocytizing photoreceptors in *Tpm1*-overexpressing microglia accelerate photoreceptor apoptosis.

To further explore the functional role of microglial *Tpm1* in RP progression, we performed signed consensus weighted gene coexpression network analysis (scWGCNA) in all *Tpm1*-expressing microglia. This approach identified four coexpression modules (Fig. 5A), with the blue module demonstrating high expression in DAM2 (Fig. 5B). Cross-module analysis revealed synergistic interaction between blue and brown modules (Fig. 5C), suggesting that hub genes in both blue and brown modules could be *Tpm1* related. KEGG pathway analysis revealed that hub genes in both blue and brown modules were primarily associated with cell cycle, P53, apoptosis, and cellular senescence pathways (Fig. 5, D to F). Moreover, we identified seven senescence drivers between DAM2 and blue and brown modules, including *Ccnb2*, *Tubb4b*, *Bnip3*, *Ctsd*, *Apoe*, *Igf1*, and *Tuba1c* (Fig. 5G), which were significantly up-regulated in *Tpm1*-overexpressing microglia (*P* < 0.05; Fig. 5H). Furthermore, component enrichment of senescence-associated secretory phenotype (SASP) in DAM2 was significantly up-regulated [Normalized Enrichment Score (NES) = 1.838, *P* = 0.004; Fig. 5I]. Together, these data suggest that accumulation of microglial *Tpm1* may result in microglial senescence. Evidently, we observed that transcriptomic levels of senescent phenotypes including *Ccnb1* (20.3%↑), *Ccnb2* (26.0%↑), *Ctsd* (22.4%↑), *Apoe* (160.7%↑), *P21* (17.0%↑), *Cdk1* (32.0%↑), *Tubb4b* (34.2%↑), *Tuba1c* (46.7%↑), *Bnip3* (12.1%↑), and *Igf1* (92.0%↑) were significantly up-regulated in microglia transfected with *Tpm1* plasmid (*P* < 0.05 versus control plasmid; Fig. 5J). In line with in vitro findings, these senescence biomarkers surge (10.0 to 58.1%↑) was observed in rd10 retinas following AAV-*Cx3cr1-Tpm1*-EGFP treatment (*P* < 0.05 versus AAV-*Cx3cr1*-EGFP controls; Fig. 5K). Together, these results indicate that *Tpm1* accumulation drives microglial senescence, with SASP components acting as key mediators of RP progression.

Consistent with established senescence-associated phagocytic decline (*21*–*23*), *Tpm1*-overexpressing microglia exhibited phagocytic suppression. The transcriptomic levels of phagocytic genes *Cd14* (37.4%↓) and *Axl* (31.2%↓) were significantly reduced in rd10 retinas treated with AAV-*Cx3cr1-Tpm1*-EGFP (*P* < 0.0001 versus AAV-*Cx3cr1*-EGFP controls; Fig. 5L). Consistently, the phagocytic receptors *Itgb1* (6.91%↓), *Cd14* (22.5%↓), and *Axl* (17.5%↓) were markedly down-regulated in microglia transfected with *Tpm1* plasmid (*P* < 0.05 versus control plasmid; fig. S5A). In addition, we observed that *Tpm1*-overexpressing microglia showed spatial engulfment impairment. By coculture of isolated retinal cells from P25 rd10 mice with microglia transfected with *Tpm1* plasmid, we observed that *Tpm1* up-regulation in microglia significantly decreased clearance of apoptotic rod (31.3%↓) and cone (35.9%↓) photoreceptors (*P* < 0.05 versus control plasmid; Fig. 5M).

Collectively, these findings suggest that *Tpm1* up-regulation in microglia triggers cellular senescence, which further compromised microglial phagocytosis for clearing apoptotic photoreceptors, subsequently leading to debris accumulation and subsequent neuroinflammation amplification (Fig. 5N), ultimately contributing to photoreceptor cell death in RP.

### Activator protein-1 signaling mediates *Tpm1*-driven neuroinflammatory cascades via MAPK kinase/extracellular signal–regulated kinase 3–dependent MAPK signaling in RP

**Fig. 6.**
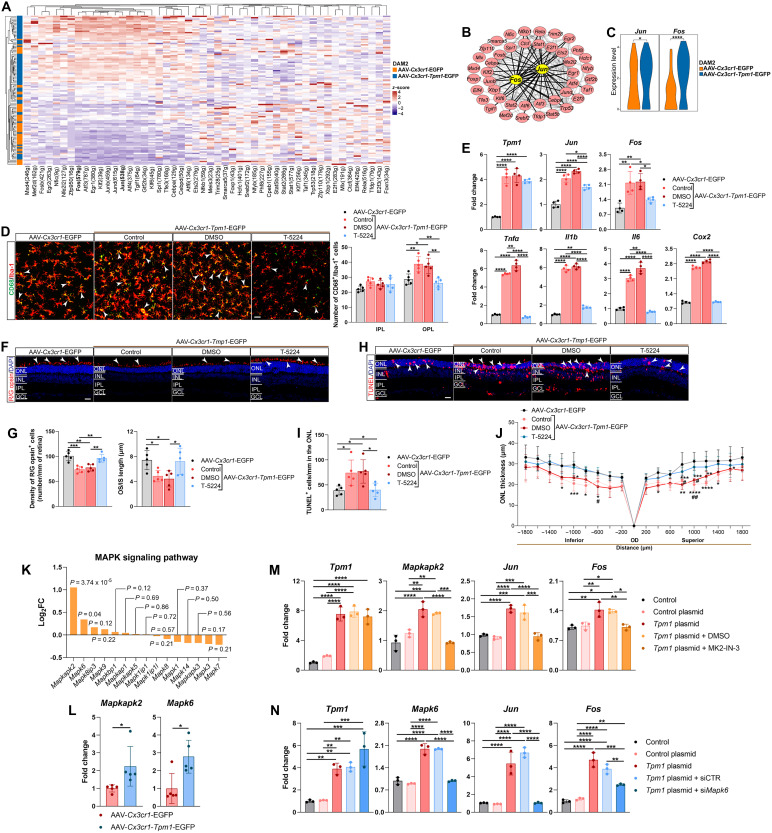
AP-1 mediates *Tpm1*-driven neuroinflammation via MK2/ERK3-dependent MAPK signaling in RP. (**A**) Heatmap of top 50 TFs in microglia from P25 rd10 retinas treated with AAV-*Cx3cr1-Tpm1*-EGFP or AAV-*Cx3cr1*-EGFP. (**B**) Protein-protein interaction (PPI) network of these TFs. (**C**) Violin plots of *Jun* and *Fos* expression in microglia. (**D**) Retinal wholemounts stained for CD68 and Iba-1, with quantification of CD68^+^ cells in OPL/IPL from P25 rd10 retinas treated with AAV-*Cx3cr1*-*Tpm1*-EGFP or AAV-*Cx3cr1*-EGFP followed by T-5224 or DMSO treatment (*n* = 5 mice per group). White arrowheads indicate CD68^+^ microglia. Four regions (638.9 × 638.9 μm, at 200 μm and 1 mm from optic nerve head) per retina were imaged along dorsal and ventral axes. (**E**) qPCR analysis of *Tpm1*, *Jun*, *Fos*, and proinflammatory cytokines (*n* = 4 mice per group). (**F** and **G**) Retinal sections stained with R/G opsin antibody (F) and quantification of R/G opsin^+^ cells and OS/IS length (G) (*n* = 5 mice per group). White arrowheads indicate R/G opsin^+^ cells. (**H** and **I**) TUNEL staining (H) and quantification of TUNEL^+^ cells in the ONL (I) (*n* = 5 mice per group). White arrowheads show TUNEL^+^ cells in the ONL. (**J**) Spider plots showing ONL thickness measured from the OD (*n* = 5 mice per group). (**K**) MAPK pathway expression in DAM2 microglia. (**L**) qPCR analysis of *Mapkapk2* and *Mapk6* in P25 rd10 retinas (*n* = 5 mice per group). (**M** and **N**) qPCR analysis of *Tpm1*, *Mapkapk2*, *Mapk6*, *Jun*, and *Fos* in BV2 microglia transfected with *Tpm1*-overexpressing plasmid and treated with MK2-IN-3 (M) or si*Mapk6* (N) (*n* = 3 independent experiments). Scale bars, 20 μm [(D), (F), and (H)]. Images [(F) and (H)] were acquired from three standardized regions at 100 μm, 1 mm, and 1.8 mm from the optic nerve head along dorsal and ventral axes. Data are mean ± SEM. Statistics: unpaired two-tailed Student’s *t* tests [(C) and (L)] and one-way [(D), (E), (G) to (I), (M), and (N)] or two-way (J) ANOVA with Tukey’s multiple-comparison test (*P < 0.05, **P < 0.01, ***P < 0.001, and ****P < 0.0001; ^#^*P* < 0.05 and ^##^*P* < 0.01).

To further explore how *Tpm1* modulates microglial dysfunction in RP, we performed SCENIC analysis among microglia isolated from rd10 retinas treated with AAV-*Cx3cr1-Tpm1*-EGFP or AAV-*Cx3cr1*-EGFP to identify potential transcription factors (TFs) associated with *Tpm1* function. We found that up-regulated *Tpm1* triggered distinct TF cascades: the higher regulon activities of *Fos*, *Fosb*, *Jun*, *Junb*, *Jund*, *Egr1*, *Egr2*, *Spi1*, and *Atf3* in *Tpm1*-overexpressing microglia (Fig. 6A). Notably, we observed that activator protein-1 (AP-1) (*Jun/Fos*) pathway was strongly interacted with other TFs (Fig. 6B), suggesting that AP-1 emerges as an important regulator of *Tpm1* pathology. The transcriptomic levels of *Jun* and *Fos* were significantly up-regulated in *Tpm1*-overexpressing microglia (127.25 to 184.3%↑, *P* < 0.05 versus AAV-*Cx3cr1*-EGFP controls; Fig. 6C and fig. S6A; and 72.6 to 120.3%↑, *P* < 0.001 versus control plasmid; fig. S6B). To further confirm the role of AP-1 signaling in *Tpm1*-mediated microglial dysfunction, we intravitreally injected T-5224 (a specific inhibitor of AP-1 pathway) in rd10 mice treated with AAV-*Cx3cr1-Tpm1*-EGFP. Evidently, we found that T-5224 treatment significantly inhibited reprogramming in microglia following *Tpm1* up-regulation: Cell density of CD68^+^ microglia in the OPL decreased 30.3% [*P* < 0.01 versus dimethyl sulfoxide (DMSO) control; Fig. 6D], and more ramified microglia were observed in T-5224–treated retinas (Fig. 6D). Meanwhile, we observed that T-5224 treatment significantly attenuated proinflammatory cytokines storm (*Tnf*α, 88.7%↓; *Il1b*, 71.2%↓; *Il6*, 78.5%↓; and *Cox2*, 65.1%↓, *P* < 0.0001 versus DMSO control; Fig. 6E) and SASP components (*Ccnb1*, 72.4%↓; *Ccnb2*, 48.6%↓; *Ctsd*, 34.9%↓; *Apoe*, 13.6%↓; *Cdk1*, 63.3%↓, *Tubb4b*, 20.4%↓; *Bnip3*, 13.2%↓; and *Igf1*, 29.0%↓, *P* < 0.05 versus DMSO control; fig. S6C). Together, these results demonstrate that AP-1 inhibition mitigates TPM1-induced microglial reprogramming, neuroinflammation, and cellular senescence.

In addition, AP-1 pathway inhibition significantly rescued photoreceptor apoptosis in rd10 retinas with microglial *Tpm1* overexpression. Specifically, we observed that cell density and OS/IS length of cone photoreceptors increased by 24.6 and 63.4%, respectively, in T-5224–treated retinas compared to DMSO controls (*P* < 0.05; Fig. 6, F and G). T-5224 treatment in microglial *Tpm1*–overexpressed retinas significantly decreased TUNEL^+^ cells (48.0%↓, *P* < 0.05 versus DMSO controls; Fig. 6, H and I) in the ONL and increased ONL thickness (*P* < 0.05 versus DMSO controls; Fig. 6J). Together, these findings demonstrate that AP-1 is a critical transcriptional effector of *Tpm1*-driven neurodegeneration and that activation of the *Tpm1*/AP-1 pathway contributes to neuroinflammation and photoreceptor loss in RP.

Prior studies have established the regulatory role of the MAPK signaling pathway in modulating AP-1 activity (*24*, *25*). Our single-cell transcriptomic profiling identified a pronounced up-regulation of MAPK signaling in DAM2 microglial cluster from rd10 retinas treated with AAV-*Cx3cr1-Tpm1*-EGFP (Fig. 4G), suggesting that *Tpm1* amplification in microglia potentiates this pathway in RP. We found that *Mapkapk2* (MK2) and *Mapk6* [extracellular signal–regulated kinase 3 (ERK3)]—two key MAPK components—were selectively elevated in DAM2 cluster following *Tpm1* overexpression (Fig. 6K). This finding was corroborated in rd10 retinas treated with *AAV-Cx3cr1-Tpm1-EGFP*, where both genes exhibited marked transcriptional activation (Fig. 6L), implicating MK2 and ERK3 as critical mediators of AP-1 hyperactivity in *Tpm1*-driven retinal pathology. Mechanistically, in vitro assays using BV2 microglia revealed that *Tpm1* overexpression robustly enhanced *Jun* and *Fos* transcription (Fig. 6, M and N). Notably, this effect was abolished by pharmacological inhibition of MK2 (MK2-IN-3) or siRNA-mediated *Mapk6* knockdown (Fig. 6, M and N). Collectively, our results unveil a critical axis wherein *Tpm1* amplification in microglia drives AP-1 hyperactivation via MK2/ERK3-dependent MAPK signaling, providing the first evidence of this pathway’s role in RP.

### *Tpm1* modulates *Apoe/Fabp5*-mediated lipid dysregulation in microglia in RP

**Fig. 7.**
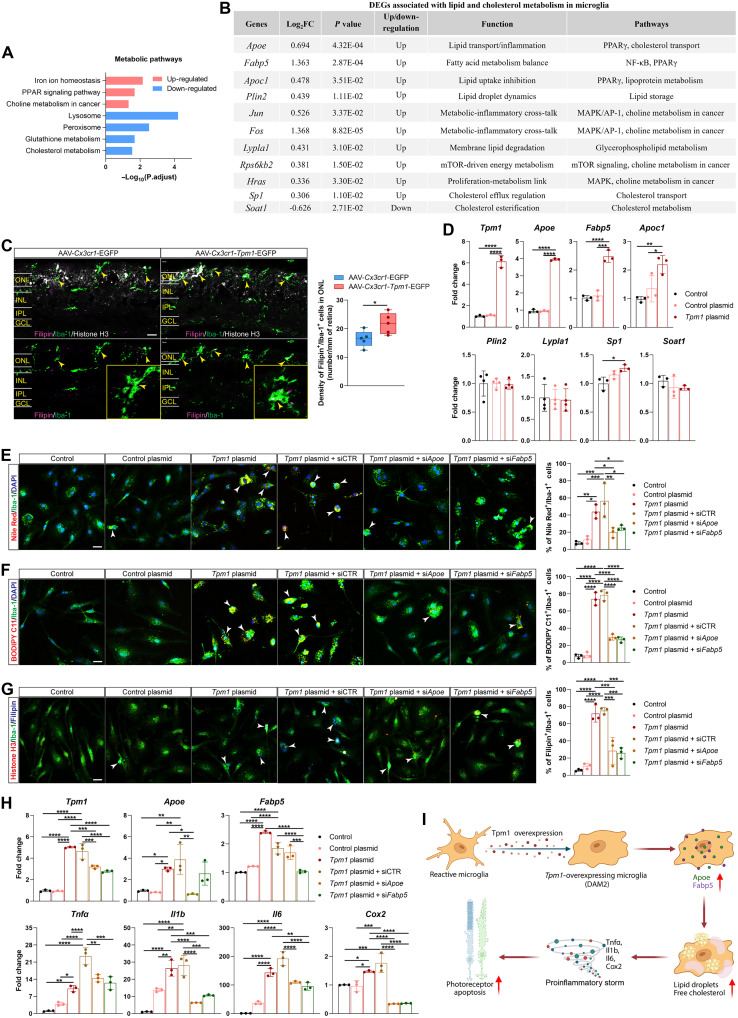
*Tpm1* modulates *Apoe/Fabp5*-mediated lipid dysregulation in microglia in RP. (**A**) Metabolism pathway enrichment analysis of DEGs in DAM2 microglial cluster. (**B**) Lipid/cholesterol metabolism–related DEGs in DAM2. mTOR, mechanistic target of rapamycin. (**C**) Retinal section stained with Filipin (free cholesterol; magenta), Iba-1 (microglia; green), and Histone H3 (nuclei; white) antibodies and quantification of Filipin^+^ microglia in the ONL of the retinas from rd10 retinas administrated with AAV-*Cx3cr1-Tpm1*-EGFP or control vector (*n* = 5 mice per group). Yellow arrowheads indicate Filipin^+^ microglia. Scale bar, 20 μm. For image acquiring, three views in each retinal section at 100 μm (central), 1 mm (middle), and 1.8 mm (peripheral) from the optic nerve head along the dorsal and ventral directions were captured. (**D**) qPCR validation of *Tpm1* and lipid metabolism regulators (*Apoe*, *Fabp5*, *Apoc1*, *Plin2*, *Lypla1*, *Sp1*, and *Soat1*) in primary microglia transfected with *Tpm1* plasmid versus control (*n* = 3 independent experiments). (**E** to **G**) Fluorescent colocalization analysis of lipid and cholesterol accumulation (Nile Red: lipid droplets; BODIPY C11: neutral lipids; Filipin: free cholesterol) in microglia following *Tpm1* overexpression and *Apoe/Fabp5* knockdown. Cell percentages of Nile Red^+^, BODIPY C11^+^, or Filipin^+^ microglia were quantified (*n* = 3 independent experiments). Scale bars, 20 μm. (**H**) Transcriptional profiling of inflammatory mediators in dual-modified microglia (*n* = 3 independent experiments). (**I**) Schematic model: *Tpm1* up-regulation drives *Apoe/Fabp5*-dependent lipid accumulation in microglia, promoting neuroinflammatory responses and photoreceptor degeneration in RP. Data represent means ± SEMs. Statistical significance determined using unpaired two-tailed Student’s *t* test (C) or one-way ANOVA with Tukey’s multiple-comparison test [(D) to (H)] (*P < 0.05, **P < 0.01, ***P < 0.001, and ****P < 0.0001).

Single-cell transcriptomic profiling revealed that *Tpm1*-overexpressing DAM2 microglia in AAV-*Cx3cr1-Tpm1*-EGFP–treated rd10 retinas exhibited marked metabolic reprogramming, characterized by enhanced cellular amide metabolism, mitochondrial complex assembly, and ribosomal biogenesis compared to vector controls (fig. S4C). Metabolic pathway enrichment analysis identified significant activation of iron ion homeostasis (adjusted *P* = 0.007), peroxisome proliferator–activated receptor (PPAR) signaling (adjusted *P* = 0.021), and choline metabolism in cancer (adjusted *P* = 0.047) in these cells (Fig. 7A). Notably, lipid/cholesterol metabolism–related DEGs including *Apoe* (*26*), *Fabp5* (*27*), *Apoc1* (*28*), *Plin2* (*29*), *Jun* (*30*, *31*), *Fos* (*30*, *31*), *Lypla1* (*32*), *Rps6kb2* (*33*), *Hras* (*34*), and *Sp1* (*35*) were significantly elevated in *Tpm1*-overexpressed DAM2 microglia from AAV-*Cx3cr1-Tpm1*-EGFP–treated rd10 retinas (Fig. 7B), suggesting that microglial *Tpm1* drives lipid/cholesterol accumulation in RP. Consistent with the transcriptomic analysis, *Tpm1*-overexpressing retinas showed higher expression levels of *Apoe* (60.4%↑), *Fabp5* (40.8%↑), *Plin2* (23.2%↑), and *Lypla1* (12.2%↑) (fig. S7A). Quantitative morphometric analysis revealed significant lipid droplet accumulation in *Tpm1*-overexpressing microglia, as evidenced by significant increase of Nile Red^+^ lipid droplet (30.9%↑), BODIPY C11^+^ neutral lipids (37.6%↑), and Filipin^+^ free cholesterol (32.3%↑) in microglia in the ONL of rd10 retinas treated with AAV-*Cx3cr1-Tpm1*-EGFP (Fig. 7C and fig. S7, B and C).

Mechanistic studies in primary microglia demonstrated that *Tpm1* transfection induced 3.2-fold *Apoe* (*P* < 0.0001) and 1.3-fold *Fabp5* (*P* < 0.001) up-regulation (Fig. 7D). Lipidomic profiling showed that *Tpm1* up-regulation in microglia resulted in 3.2- to 7.6-fold increase of intracellular lipid droplets and 5.7-fold elevation of free cholesterol, which were inhibited by *Apoe/Fabp5* suppression (Fig. 7, E to G). These results place the *Apoe/Fabp5* pathway downstream of *Tpm1* in regulating microglial lipid homeostasis. Crucially, *Tpm1*-mediated neuroinflammation—evidenced by significant elevation of *Tnf*α (158%↑), *Il1b* (92.6%↑), *Il6* (287%↑), and *Cox2* (53.2%↑)—was attenuated by 38.4 to 80.8% following *Apoe/Fabp5* knockdown (Fig. 7H).

Together these findings establish a critical *Tpm1-Apoe/Fabp5* regulatory axis driving microglial lipotoxicity in photoreceptor degeneration (Fig. 7I). This pathway represents a previously unrecognized mechanism linking cytoskeletal remodeling (via *Tpm1*) to lipid-mediated neuroinflammation, highlighting a promising therapeutic entry point for modulating microglial metabolic states in RP.

### Spatiotemporal propagation of *Tpm1*-driven neuroinflammation through *Lgals9/Cd45* signaling axis in RP

**Fig. 8.**
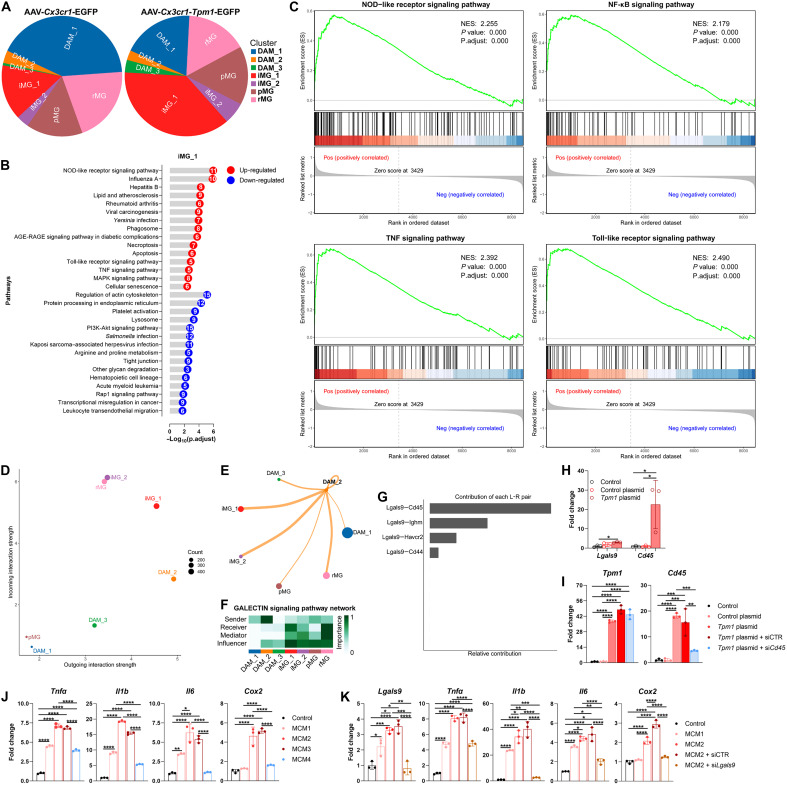
Spatiotemporal propagation of *Tpm1*-driven neuroinflammation through *Lgals9/Cd45* signaling axis in RP. (**A**) The pie chart showing cell ratio in different clusters of microglia isolated from P25 rd10 retinas treated with AAV-*Cx3cr1-Tpm1*-EGFP or AAV-*Cx3cr1*-EGFP. (**B**) Kyoto Encyclopedia of Genes and Genomes (KEGG) pathway analysis of DEGs in iMG_1 cluster. PI3K, phosphatidylinositol 3-kinase. (**C**) GSEA analysis of NOD-like receptor, NF-κB, TNF, and Toll-like receptor signaling pathways in iMG_1. (**D**) Scatter plot showing both incoming and outgoing interaction strength in each microglial cluster. (**E**) Circle plot depicting the directed interaction between DAM2 and other microglial clusters. (**F**) Heatmap showing the importance of four centrality indicators of the GALECTIN signaling pathway network in different microglial clusters. (**G**) Contribution of each ligand-receptor (LR) pair in GALECTIN signaling pathway. (**H**) qPCR analysis of expression of *Lgals9* and *Cd45* in microglia isolated from 6-week-old C57BL/6J mice and transfected with *Tpm1* plasmid or control plasmid. (**I**) qPCR analysis of *Tpm1* and *Cd45* expression in microglia isolated from 6-week-old C57BL/6J mice and transfected with both *Tpm1* plasmid and si*Cd45*, both *Tpm1* plasmid and siCTR, or only *Tpm1* plasmid or control plasmid. (**J**) qPCR analysis of proinflammatory cytokines in microglia isolated from 6-week-old C57BL/6J mice and treated with MCM1, MCM2, MCM3, and MCM4. (**K**) qPCR analysis of *Lgals9* and proinflammatory cytokines in microglia isolated from 6-week-old C57BL/6J mice and transfected with si*Lgals9* or siCTR followed by treatment with MCM1 or MCM2. The results shown represent three independent experiments. The data are presented as the means ± SEMs and were analyzed via one-way ANOVA with Tukey’s multiple-comparison test [(H) to (K)] (*P < 0.05, **P < 0.01, ***P < 0.001, and ****P < 0.0001).

Single-cell spatial mapping revealed that *Tpm1*-overexpressing DAM2 microglia (Fig. 4A) drive expansion of adjacent iMG_1 population (1.51-fold increase versus AAV-*Cx3cr1*-EGFP controls; Fig. 8A). Transcriptomic profiling of iMG_1 microglia demonstrated that cytokine storm reprogramming was significantly up-regulated (fig. S8, A and B). In particular, inflammation-associated pathways including NOD-like receptor signaling pathway (NES = 2.255), NF-κB signaling pathway (NES = 2.179), TNF signaling pathway (NES = 2.392), and Toll-like receptor signaling pathway (NES = 2.490) were significantly up-regulated in iMG_1 from AAV-*Cx3cr1-Tpm1*-EGFP–treated rd10 retinas (*P* < 0.0001 versus AAV-*Cx3cr1*-EGFP controls; Fig. 8, B and C). These spatial and transcriptional features suggest direct intermicroglial communications linking *Tpm1*-high DAM2 microglia to adjacent iMG_1 cells.

To interrogate this interaction, CellChat analysis identified *Tpm1*-overexpressing DAM2 as a dominant signaling “sender,” exhibiting the highest outgoing interaction strength (Fig. 8D) with preferential targeting of its spatially proximal iMG_1 population (Fig. 8E). Ligand-receptor (LR) pair analysis unveiled multiple LR pairs including *Lgals9-Ptprc* (*Cd45*), *Lgals9-Ighm*, and *Ccl4/Ccl3-Ccr5*, which were highly probed between DAM2 and other microglial clusters (fig. S8C). Notably, GALECTIN signaling pathway, which was primarily sent by *Tpm1*-overexpressing DAM2, strongly influenced its neighboring iMG_1 microglia (Fig. 8F). Within this pathway, the *Lgals9-Cd45* pair emerged as the principal contributor (Fig. 8G). These data suggest that *Lgals9/Cd45* axis could be an important regulator of *Tpm1*-dependent inflammatory propagation.

Unexpectedly, we observed that *Cd45* was significantly up-regulated in *Tpm1*-overexpressing microglia (247.2%↑, *P* < 0.05 versus control plasmid; Fig. 8H), suggesting that microglial *Tpm1* up-regulation induces *Cd45* surge. When stimulating microglia isolated from C57BL/6J mice with cell supernatants collected from microglia transfected with both *Tpm1* plasmid and si*Cd45* (MCM4), both *Tpm1* plasmid and siCTR (MCM3), and only *Tpm1* plasmid (MCM2) or control plasmid (MCM1) (efficiency: *Tpm1*, 33.2-fold↑; *Cd45*, 70.4%↓, *P* < 0.01; Fig. 8I), we found that MCM3 or MCM2 treatment significantly increased proinflammatory cascades in microglia, which were significantly reversed following MCM4 treatment (*Tnf*α, 42.3 to 44.4%↓; *Il1b*, 65.3 to 72.3%↓; *Il6*, 79.4 to 82.3%↓; *Cox2*, 65.0 to 68.6%↓, versus MCM3 or MCM2, *P* < 0.0001; Fig. 8J). These results support a model in which *Cd45* is required for TPM1-driven inflammatory signaling that spreads to neighboring microglia. To directly verify the ligand component of the predicted axis, we silenced *Lgals9* in recipient microglia (knockdown efficiency: 77.0%↓) before stimulation with MCM2 or MCM1. *Lgals9* knockdown in microglia significantly hampered proinflammatory surges induced by MCM2 treatment (*Tnf*α, 40.3 to 41.2%↓; *Il1b*, 92.5 to 93.6%↓; *Il6*, 53.9 to 58.2%↓; *Cox2*, 39.9 to 57.5%↓, *P* < 0.001 versus MCM2 or MCM2 + siCTR; Fig. 8K), demonstrating that *Lgals9* signaling is necessary for propagation of *Tpm1*-initiated inflammatory activation. In addition, we observed that MCM2 treatment increased SASP signatures in recipient microglia, which were markedly inhibited following *Lgals9* suppression (*Ccnb2*, 40.6 to 49.0%↓; *Ctsd*, 8.9 to 16.1%↓; *P21*, 31.5 to 46.6%↓; *Cdk1*, 16.1 to 18.9%↓; *Bnip3*, 65.0 to 67.0%↓; *Igf1*, 25.6%↓, *P* < 0.05 versus MCM2 or MCM2 + siCTR; fig. S8D). Collectively, these findings indicate that *Tpm1*-overexpressing microglia propagate spatial inflammation and SASP-associated programs to neighboring microglia through an *Lgals9/Cd45* signaling axis and that disrupting this cross-talk may represent a tractable strategy to limit sustained proinflammatory response in RP.

**Fig. 9.**
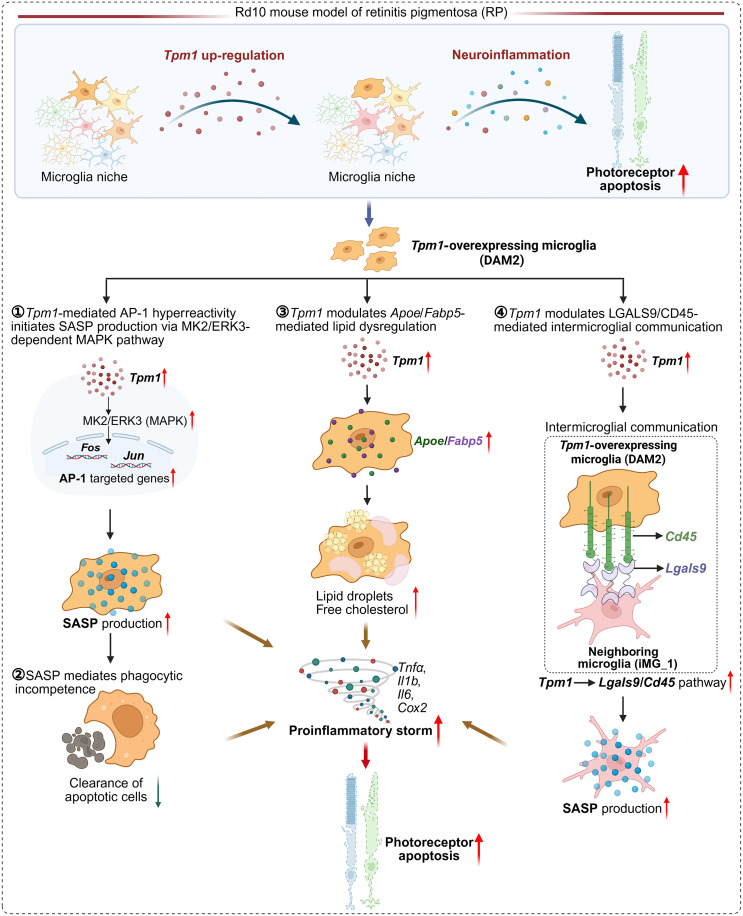
Schematic illustration showing the role of microglial *Tpm1* in regulating neuroinflammation and photoreceptor degeneration in RP. This study demonstrates that TPM1-overexpressing DAM drive neuroinflammation and photoreceptor degeneration through four interconnected mechanisms: (i) TPM1-mediated AP-1 hyperactivation promotes SASP production via MK2/ERK3-dependent MAPK signaling; (ii) SASP subsequently impairs phagocytic capacity; (iii) *Apoe/Fabp5*-mediated lipid dysregulation leads to pathogenic lipid droplet accumulation and cholesterol crystallization; and (iv) LGALS9/CD45-dependent cross-talk between TPM1-expressing microglia and their neighbors sustains spatially propagating inflammation.

In conclusion, our study establishes TPM1 as a pivotal orchestrator of neuroinflammatory propagation in RP, revealing its dual role in dictating cytoskeletal remodeling and immunometabolic reprogramming within microglia. Through multimodal interrogation of the rd10 model, we demonstrate that TPM1-overexpressing DAM regulate neuroinflammation and photoreceptor degeneration via four interlinked mechanisms: (i) TPM1-mediated AP-1 hyperreactivity initiates SASP production via MK2/ERK3-dependent MAPK signaling, (ii) SASP subsequently mediated reduced phagocytosis, (iii) *Apoe/Fabp5*-mediated lipid dysregulation inducing pathogenic lipid droplet accumulation with cholesterol crystallization, and (iv) the cross-talk between *Tpm1*-expressing microglia and its neighboring microglia governed by the LGALS9/CD45 axis accelerates the sustained spatial inflammation spreading (Fig. 9). These findings decode a long-standing paradox in retinal neurodegeneration research—how focal photoreceptor apoptosis propagates global retinal neuroinflammation, providing the first evidence of actin dynamics–immunometabolism coupling in degenerative cascades. This mechanistic framework extends beyond RP, offering insights into neuroinflammatory regulation in AD and PD where TPM1 dysregulation is implicated (*11*, *13*, *14*, *17*). The conserved nature of these pathways positions TPM1 network inhibitors as potential pan-degeneration therapeutics, bridging the gap between ocular and CNS neurodegeneration research.

## DISCUSSION

Our study establishes TPM1 as a central orchestrator of spatially organized neuroinflammation in retinal degeneration, revealing its dual role in cytoskeletal remodeling and immunometabolic reprogramming. Through integrated transcriptomic-proteomic-lipidomic profiling of the rd10 RP model, we demonstrate that microglial TPM1 overexpression drives neurodegenerative progression via four interlocked mechanisms: AP-1–mediated transcriptional ignition, SASP-dependent reduced phagocytosis, *Apoe/Fabp5*-driven lipid dysregulation, and LGALS9/CD45-mediated intercellular communication causing the sustained global inflammation spreading. These findings fundamentally advance our understanding of neuroinflammatory propagation in inherited retinal diseases while exposing some therapeutic targets.

This study establishes three paradigm-shifting insights. First, we provide the inaugural evidence of cytoskeleton-immunometabolism coupling in retinal degeneration, revealing how TPM1-mediated actin polymerization [actin monomers (G-actin)↑ and filaments (F-actin)↑] (*36*–*38*) sustains AP-1 transcriptional hyperactivity through initiating *Tpm1*/AP-1 signaling pathway—a mechanism previously characterized in cancer metastasis (*39*, *40*) but never reported in neurodegenerative contexts. Second, we identify LGALS9/CD45 as the first-known intermicroglial SASP propagation conduit, extending recent reports of galectin-mediated immune regulation in allergy (*41*, *42*), arthritis (*42*, *43*), and systematic sclerosis (*44*) to retinal neurodegeneration. Third, our discovery that lipid accumulation (Nile Red^+^ lipid droplets↑ and BODIPY C11^+^ neutral lipid↑) and cholesterol crystallization (Filipin^+^ inclusions↑) in *Tpm1*-overexpressing microglia fuel inflammatory amplification establishes lipid phase transition as a pathogenic mechanism in RP, paralleling findings in nonalcoholic steatohepatitis (*45*) and atherosclerosis (*46*) but with distinct microglial pathophysiology. These mechanisms collectively address the longstanding mystery of how localized photoreceptor apoptosis triggers pan-retinal inflammation. Our transcriptomics and spatial proteomic data reveal that *Tpm1*-overexpressing DAM microglia establish inflammatory niche (*Tnf*α↑, *Il1b*↑, *Il6*↑, and *Cox2*↑), creating self-reinforcing degenerative loops that accelerate photoreceptor cell loss.

Current therapeutic strategies for RP remain predominantly focused on genetic correction (*47*, *48*), photoreceptor replacement (*49*), and neuroprotective approaches (*47*, *50*), with limited consideration of the inflammatory microenvironment—a critical oversight given our demonstration that microglial TPM1 drives ⁓55% of degenerative inflammation in rd10 retinas. While prior studies established neuroinflammation as a pathological amplifier in RP (*7*), the absence of defined molecular targets has hindered therapeutic development. Our work fundamentally addresses this gap by identifying the TPM1-LGALS9/CD45 axis as a potential regulatory axis driving inflammation propagation. Notably, we demonstrate that targeted suppression of either CD45 in *Tpm1*-overexpressing DAM2 microglia or LGALS9 in neighboring iMG_1 microglia markedly reduces inflammatory spread, establishing this axis as the critical signaling conduit in RP-associated neuroinflammation. This finding contrasts with previous attempts to broadly inhibit microglial activation using colony-stimulating factor 1 receptor (CSF1R) antagonists (*51*, *52*), which indiscriminately deplete both pathogenic and homeostatic microglial subsets. In addition, emerging galectin inhibitors such as LYT-200 (*53*) and TD139 (*53*), currently in phase I/II trails for metastatic solid tumors (*53*) and idiopathic pulmonary fibrosis (*53*), could be repurposed for RP given conserved LGALS9 structures across tissues.

While microglial activation has been established as a pathological hallmark of RP (*6*, *10*), previous investigations predominantly focused on downstream inflammatory mediators [TNF-α, interferon-γ, NF-κB, C-C Motif Chemokine Ligand 2 and 5 (CCL2/5), and component of complement 1q (C1q)] (*5*, *7*, *54*), leaving upstream regulatory mechanisms unresolved. Our work resolves this critical knowledge gap by identifying TPM1 as the molecular linchpin connecting genetic susceptibility [phosphodiesterase 6B (PDE6β) mutation in *rd10*] with environmental stressors (oxidized lipid accumulation), thereby mechanistically unifying the “two-hit” hypothesis of RP progression (*55*, *56*). Specifically, we demonstrate that *Tpm1*-overexpressing microglia establish a self-perpetuating cycle of lipid dysregulation—characterized by aberrant lipid droplet biogenesis and pathogenic cholesterol crystallization—which directly exacerbates photoreceptor toxicity beyond primary genetic insults. This discovery implicates transitioning from a neuron-centric to microglia-driven disease model, where cytoskeletal remodeling modulates immunometabolic cross-talk. Given TPM1’s established role in AD-associated microglial dystrophy (*11*, *15*), our findings suggest a unified pathway for neuroinflammatory propagation across degenerative disorders.

While we establish TPM1’s centrality in RP-associated neuroinflammation, three limitations should warrant consideration in the future. First, while rd10 model recapitulates key RP features (*19*, *57*), human RP exhibits greater genetic heterogeneity—future studies should validate findings in patient-derived microglia organoids. Second, our temporal analysis (P16 to P25) captures early and peak degeneration phases but not trace TPM1 dynamics in development stage. Third, although we demonstrate LGALS9-CD45 binding in driving *Tpm1*-mediated spatial inflammation propagation, it still remains unclear whether its downstream signaling cascade [c-Jun N-terminal kinase and P38 pathways (*58*)] might be involved, which requires further dissection in the future.

By mapping the TPM1-LGALS9/CD45 axis as a critical factor of neuroinflammatory propagation, this work redefines RP pathogenesis from a cell-autonomous photoreceptor disorder to a spatially coordinated inflammatory milieu. Our mechanistic dissection provides both a framework for understanding neuroglial cross-talk in inherited retinopathies and a new insight for developing targeted immunotherapies. The conserved nature of these pathways across neurodegenerative diseases suggests broader implications for treating conditions where microglial activation drives progression, from geographic atrophy to Parkinson’s dementia.

## MATERIALS AND METHODS

### In vivo experiments

#### Mice

C57BL/6J (stock no: 000664) and rd10 (stock no: 004297) mice were obtained from the Jackson Laboratory. All mice were housed in a 12-hour light/dark cycle with water and food ad libitum and maintained at the Centralised Animal Facilities, The Hong Kong Polytechnic University. All experimental procedures were approved by the Animal Subjects Ethics Sub-committee (ASESC) of The Hong Kong Polytechnic University and conducted in accordance with the Association for Research in Vision and Ophthalmology (ARVO) statement for the use of animals (the approval number of ASESC: 23-24/1029-SO-R-HMRF). Mice were excluded from the study if they exhibited predefined ocular abnormalities, including atrophy due to malnutrition (typically caused by inadequate maternal care in larger litters) or cataract formation resulting from injection injury or other genetic factors. Eleven rd10 mice were excluded on these grounds, while no C57BL/6J mice were excluded, yielding a final cohort of 108 rd10 and 42 C57BL/6J mice.

#### Intravitreal siRNA injections

To study the role of *Tpm1* in microglia dysfunction and photoreceptor cell death in RP, we injected intravitreally si*Tpm1* and siCTR (Thermo Fisher Scientific, Hong Kong) into rd10 mouse eyes at P16 as previously described (*16*). Briefly, rd10 mice were systemically anesthetized through intraperitoneal injection of ketamine hydrochloride (100 mg/kg) and xylazine hydrochloride (50 mg/kg) cocktail and then properly positioned under a stereomicroscope for precise ocular manipulation. A surgical incision was carefully created in the superior nasal scleral region using a sterile 31-gauge hypodermic needle. Subsequently, a borosilicate glass microinjection pipette containing 1 μl of either 100 μM si*Tpm1* or siCTR solution was vertically introduced into the vitreous chamber through the established surgical portal. The therapeutic agent was administered via slow manual injection (30-s duration) to ensure optimal intraocular distribution. This intravitreal injection protocol was repeated three times at 72-hour intervals over a 9-day treatment period. Postoperative care included topical application of ophthalmic antibiotic ointment (neomycin–polymyxin B–bacitracin) to prevent microbial contamination. *Tpm1* knockdown efficacy was quantitatively assessed through comparative analysis using quantitative real-time polymerase chain reaction (qRT-PCR). We excluded the potential effects of intravitreal injection procedure itself on microgliosis and retinal structure in C57BL/6J mice under the same injection regimen (fig. S9).

#### Subretinal injection of AAVs

To investigate the functional role of microglial *Tpm1* in photoreceptor degeneration in RP, neonatal rd10 mice (P0 to P1) underwent subretinal administration of a recombinant AAV serotype 5 (AAV5) carrying the *Tpm1* gene under the control of the microglia-specific *Cx3cr1* promoter (*16*, *59*–*61*), with a fused enhanced green fluorescent protein (EGFP) reporter (AAV-*Cx3cr1-Tpm1*-EGFP; GeneChem, China). Neonatal rd10 pups were subjected to hypothermic anesthesia via surface cooling on crushed ice for 3 to 5 min, with continuous monitoring of respiratory rate and pedal reflex to ensure adequate sedation. A precise keratotomy was performed along the fused limbal junction using a sterile 30-gauge beveled needle (BD Biosciences, USA) to establish surgical access to the subretinal space. A total volume of 0.4 μl of AAV5 suspension [titer: 1 × 10^13^ viral genomes (vg)/ml] was aspirated into a polished glass microinjection needle (World Precision Instruments, USA) pretreated with a micropipette beveller (Narishige, Japan). The needle was advanced transsclerally into the subretinal compartment, followed by controlled infusion at a rate of 10 nl/s using a programmable microinjection system (R-480 Nanoliter Pump, RWD Life Science, USA) to minimize mechanical trauma. Following injection, the ocular surface was disinfected with 70% ethanol, and pups were transferred to a heating pad with 37°C until full restoration of normothermia and locomotor activity. *Tpm1* overexpression in retinal microglia was confirmed via retinal section immunofluorescence and quantitative assessment of colocalized TPM1^+^/EGFP^+^ cells in the retina.

#### T-5224 treatment

To determine whether AP-1 signaling mediated microglial *Tpm1*–driven photoreceptor degeneration in RP, a pharmacological inhibition paradigm was implemented. Neonatal rd10 mice (P0 to P1) first received subretinal injections of either AAV5-*Cx3cr1-Tpm1*-EGFP (experimental group) or AAV5-*Cx3cr1*-EGFP (vector control; GeneChem, China) as previously described. At P16, these animals subsequently underwent serial intravitreal administrations of T-5224 [50 mM in 10% DMSO; MedChemExpress (MCE), USA], a selective AP-1 transcriptional activity inhibitor, or vehicle control [10% DMSO in phosphate-buffered saline (PBS)], delivered in triplicate at 72-hour intervals (P16, P19, and P22). T-5224 working solution (50 mM) was freshly prepared by dissolving lyophilized compound in 10% DMSO (Sigma-Aldrich, USA) diluted with PBS (pH 7.4). Following the established microinjection methodology (R-480 Nanoliter Pump, RWD Life Science), 1 μl of therapeutic agent or vehicle was administered into the vitreous chamber, with postinjection antibiotic prophylaxis as previously detailed. Transcriptional suppression of AP-1 signaling was quantitatively assessed 72 hours post–final injection (P25) via qPCR analysis.

#### Immunocytochemistry and confocal imaging

Following enucleation, ocular globes were immediately immersed in ice-cold PBS (pH 7.4). Retinal tissues were meticulously dissected from surrounding vitreous humor and scleral layers under stereomicroscopic guidance (ZEISS, Germany). Isolated retinas underwent chemical fixation in 4% paraformaldehyde (PFA; Sigma-Aldrich) for 60 min at room temperature, followed by cryoprotection through graded sucrose immersion [30% (w/v) in PBS] overnight at 4°C. Retinal specimens were embedded in optimal cutting temperature compound (Tissue-Tek, Sakura) and sectioned coronally at 14-μm thickness using a cryostat microtome (Leica) maintained at −20°C. After rinsing three times with PBS, the retina sections were incubated with rat CD68 (Bio-Rad; 1:500), rabbit TPM1 (Invitrogen, 1:200; ABclonal, 1:200), rabbit red/green (R/G) opsin (Millipore; 1:250), rabbit ionized calcium-binding adapter molecule 1 (Iba-1) (Wako; 1:500) and goat Iba-1 (Wako; 1:500) in blocking buffer containing 3% normal donkey serum, 1% bovine serum albumin (BSA) and 0.3% Triton X-100 in PBS (pH 7.4), overnight. Afterward, donkey anti-rabbit Alexa Fluor 594 (Invitrogen; 1:500), donkey anti-goat Alexa Fluor 594 (Invitrogen; 1:500), donkey anti-rat Alexa Fluor 488 (Molecular Probes; 1:500), and donkey anti-rabbit Alexa Fluor 488 (Invitrogen; 1:500) were incubated for 2 hours before mounting slides with Dako fluorescence mounting medium. For wholemounted retinas, CD68 (Bio-Rad; 1:500) and Iba-1 (Wako; 1:500) were individually incubated for 24 hours, followed by incubating with donkey anti-rat Alexa Fluor 488 (Molecular Probes; 1:500) and donkey anti-rabbit Alexa Fluor 594 (Invitrogen; 1:500) for 2 hours. Fluorescence images of retinal sections and wholemounted retinas were captured by a Zeiss LSM 800 Upright Confocal Microscope (ZEISS, USA) with a pixel resolution of 1024 × 1024 and Plan-Apochromat 20×/0.8 numerical aperture (NA) objective. Z-stack images with an interval of 0.8 μm was acquired. For the quantification of the number of R/G opsin–positive cone photoreceptors and microglia density in the ONL, three areas at 100 μm (central area), 1 mm (middle), and 1.8 mm (peripheral) from the optic nerve head along the dorsal and ventral directions in each retinal section were captured. The number of R/G opsin–positive cone photoreceptors and microglia density in the ONL in each capture was manually counted. For quantification of microglial cells, four sampling areas with 638.9 μm–by–638.9 μm squares along the dorsal and ventral axes of retinal wholemounts at 200 μm and 1 mm from the optic nerve head on both sides were photographed, and the numbers of CD68^+^/Iba-1^+^ or EGFP^+^/Iba-1^+^ microglial cells were manually counted.

#### Cellular lipid staining

Retinal sections were incubated with goat Iba-1 (Wako; 1:500) in blocking buffer at 4°C overnight, followed by incubation with donkey anti-goat Alexa Fluor 488 (Invitrogen; 1:500) and Nile Red (MCE; 10 μg/ml) or BODIPY 581/591 C11 (Lipid Peroxidation Sensor) (Invitrogen; 1:1000) for 2 hours. Cellular lipid in microglia was observed by a Zeiss LSM 800 Upright Confocal Microscope (ZEISS, USA).

#### Free cholesterol staining

Retinal sections were incubated with goat Iba-1 (Wako; 1:500) and rabbit Histone H3 (MCE; 1:100) in blocking buffer at 4°C overnight, followed by incubation with donkey anti-goat Alexa Fluor 488 (Invitrogen; 1:500) and donkey anti-rabbit Alexa Fluor 594 (Invitrogen; 1:500) for 2 hours. After rinsing three times with PBS, retinal sections were incubated with glycine (1.5 mg/ml; Sigma-Aldrich) for 10 min at room temperature before staining sections with Filipin complex (0.05 mg/ml; MCE) in blocking buffer for 2 hours. Free cholesterol in microglia was visualized by a Zeiss LSM 800 Upright Confocal Microscope (ZEISS, USA).

### TUNEL assay

The TUNEL assay was conducted in accordance with our established protocol (*16*) to quantify apoptotic cells in retinal tissues. Retinal cryosections (14-μm thickness) were fixed in freshly prepared 4% PFA (Sigma-Aldrich) for 15 min at 37°C, followed by permeabilization with 1× proteinase K (Sigma-Aldrich) in PBS for 15 min at room temperature (25°C). After postfixation with 4% PFA (5 min at 37°C) and three 5-min washes with PBS (0.01 M, pH 7.4), sections were equilibrated in 100 μl of TdT reaction buffer [20 mM tris-HCl and BSA (0.25 mg/ml; pH 7.2)] for 10 min at 37°C. Subsequently, sections were incubated with TdT reaction mixture containing 50× uridine 5ʹ-(tetrahydrogen triphosphate) (EdUTP) (Click-iT EdUTP, Thermo Fisher Scientific) and recombinant TdT enzyme (15 U/μl; Thermo Fisher Scientific) in TdT buffer for 60 min at 37°C. TUNEL-positive nuclei were fluorescently labeled using the Click-iT Plus TUNEL Assay Kit (Invitrogen), with sections incubated in reaction cocktail containing Alexa Fluor 594 Picolyl Azide and 1× Reaction Buffer Additive for 30 min at 37°C protected from light. Following three rigorous PBS washes (10 min each), sections were counterstained with 4′,6-diamidino-2-phenylindole (DAPI; Sigma-Aldrich) for 15 min and mounted using Dako Fluorescent Mounting Medium (Agilent Technologies). Labeled specimens were imaged on a Zeiss LSM 800 confocal system equipped with Plan-Apochromat 20×/0.8 NA objective (Carl Zeiss Microscopy). For quantification of TUNEL-positive cells in the ONL of the retina in each image, three views in each retinal section at 100 μm (central), 1 mm (middle), and 1.8 mm (peripheral) from the optic nerve head along the dorsal and ventral directions were captured, and TUNEL^+^ nuclei within ONL were manually enumerated across six nonoverlapping fields per retinal quadrant using ImageJ v1.53t (National Institutes of Health) with Cell Counter plugin.

### Immunoblotting

Total protein extraction and immunoblotting were performed to quantify TPM1 expression levels in the retinas of rd10 mice, with glyceraldehyde-3-phosphate dehydrogenase (GAPDH) serving as an endogenous loading control. Freshly dissected retina was homogenized in ice-cold Radioimmunoprecipitation Assay (RIPA) Lysis Buffer (Abcam) supplemented with 1× Halt Protease & Phosphatase Inhibitor Cocktail (Roche). The homogenate was incubated on ice for 30 min with intermittent vortexing. Lysates were centrifuged at 10,000*g* for 15 min at 4°C. The lipid-free supernatant was collected, and protein concentration was determined using the Pierce Rapid Gold BCA Assay Kit (Thermo Fisher Scientific) with BSA standards. Equal protein aliquots (20 μg per lane) were denatured in Laemmli buffer containing 5% β-mercaptoethanol at 95°C for 5 min and resolved through 10% tris-glycine SDS–polyacrylamide gel electrophoresis gels (Mini-PROTEAN TGX, Bio-Rad) at constant voltage (80 V) for 90 min in running buffer (25 mM tris, 192 mM glycine, and 0.1% SDS). Afterward, proteins were electrophoretically transferred to 0.45-μm polyvinylidene difluoride membranes (IPFL00010, Millipore) at 100 V for 60 min in transfer buffer (25 mM tris, 192 mM glycine, and 20% methanol). Membranes were blocked with 5% BSA (Sigma-Aldrich) in TBST (tris-buffered saline with 0.1% Tween 20) for 1 hour at room temperature, followed by incubation with rabbit anti-TPM1 (1:1000; ABclonal) and mouse anti-GAPDH (1:3000; Millipore) antibodies in blocking buffer for 18 hours at 4°C. After three times of washes with TBST, membranes were incubated with species-matched horseradish peroxidase–conjugated secondary antibodies including goat anti-rabbit immunoglobulin G (IgG) (1:1000; Thermo Fisher Scientific) and goat anti-mouse IgG (1:1000, Thermo Fisher Scientific) for 2 hours at room temperature. The membrane with protein was evaluated by ChemiDoc Imaging Systems (Bio-Rad, California, USA) after incubating with SuperSignal West Pico PLUS Chemiluminescent Substrate (Invitrogen) or ECL Select Western Blotting Detection Reagent (Amersham). The optical density value of each band was measured using the ImageJ software.

### Quantitative real-time PCR

Freshly dissected retina was homogenized in 1 ml of ice-cold TransZol Up Plus reagent (TransGen Biotech) or TRIzol Reagent (Thermo Fisher Scientific). Following chloroform phase separation, RNA was precipitated with isopropanol and purified according to the manufacturer specifications. RNA integrity was verified by absorbance ratios (NanoDrop 2000, Thermo Fisher Scientific; *A*_260_/*A*_280_ = 1.8 to 2.1; *A*_260_/*A*_230_ > 2.0). High-capacity RNA-to-cDNA synthesis was performed using PrimeScript RT Master Mix (Takara Bio). Gene expression analysis was conducted with TB Green Premix Ex Taq (Takara Bio) using QuantStudio 7 Flex/5 Flex systems (Applied Biosystems). The endogenous reference gene *Gapdh* (glyceraldehyde-3-phosphate dehydrogenase) was used for normalization. Relative quantification was performed via the 2^–ΔΔ*C*t^ method using QuantStudio Design & Analysis Software v2.6. Primer sequences (table S1) were designed using NCBI Primer-BLAST.

### Electroretinogram analysis

Retinal functional assessment was performed via full-field electroretinography following established protocols from our laboratory (*15*). Briefly, mice underwent 12-hour dark adaptation in light-tight chambers with ad libitum access to food and water. Following anesthesia by intraperitoneal injection of ketamine hydrochloride (100 mg/kg; Sigma-Aldrich) and xylazine (50 mg/kg; Sigma-Aldrich), mice were put on a feedback-regulated heating platform at 37° ± 0.5°C. Mydriasis was performed by topical application of 1% tropicamide (Alcon) to both eyes, followed by continuous lubrication with 3% hydroxypropyl methylcellulose gel (GenTeal, Novartis). Electroretinogram (ERG) recording was performed using a Celeris ERG system (Diagnosys, USA) with TOUCH/TOUCH protocol. A scotopic ERG was detected at light intensities of 0.01, 0.1, 1, and 3 cd•s/m^2^, followed by photopic ERG detection at light intensities of 3 and 10 cd.s/m^2^ after 10-min light adaptation with background light intensity at 30 cd•s/m^2^. Ten sweeps were acquired with each light stimulus. The distance between the baseline and the negative peak was measured as the amplitude of ERG a-wave, and the amplitude of b-wave was calculated between the bottom of the a-wave and the top of the tallest curve. The latency was defined as the time interval between stimulus onset and the peak of a- or b-wave.

### Flow cytometry

Eyes were rapidly enucleated using curved iris scissors (Fine Science Tools) and placed in 4°C prechilled PBS. Retinal tissues were meticulously dissected from vitreous and scleral layers under a stereomicroscope (ZEISS), maintaining tissue viability through ice-cold processing. Retinas were enzymatically digested in 150 μl of papain (20 U/ml; Worthington) dissolved in Earle’s balanced salt solution (Worthington) supplemented with 12.5 μl of deoxyribonuclease I (DNase I) (2000 U/ml; Worthington) for 30 min at 37°C with gentle trituration every 10 min using a fire-polished Pasteur pipette. Cell suspensions were washed twice with 2% BSA and incubated with anti-CD16/32 antibody (1:100; eBioscience) in staining buffer for 10 min on ice to block Fc receptors. Afterward, cells were fixed with 200 μl of IC Fixation Buffer (Thermo Fisher Scientific) for 40 min at 25°C and permeabilized with 2 ml 1× Permeabilization Buffer (Thermo Fisher Scientific) for 15 min. Following centrifugation at 500*g* for 5 min, cells were incubated with goat anti–Iba-1 (1:200; Wako) and rabbit anti-TPM1 (1:150; ABclonal) in Perm/Wash Buffer for 40 min at 25°C, followed by incubation with donkey anti-goat Alexa Fluor 488 (1:250; Invitrogen) and donkey anti-rabbit Alexa Fluor 594 (1:250; Invitrogen) for another 40 min. Stained cells were resuspended in 300 μl of 2% BSA and filtered through 35-μm cell strainers (Falcon). Data acquisition was performed on a BD FACSAria III (BD Biosciences) equipped with 488-nm (Alexa Fluor 488) and 561-nm (Alexa Fluor 594) lasers. Compensated FCS files were analyzed using FlowJo v10.8.1 (BD Biosciences).

### Fluorescence-activated cell sorting

Retinal tissues from rd10 mice at P16 to P25 were dissociated using the protocol described in the previous section. Single-cell suspensions were first incubated with anti-CD16/32 antibody (1:100 dilution; clone 93, eBioscience) in PBS-based staining buffer for 10 min at 4°C to block Fc receptors, followed by staining with fluorescein isothiocyanate–conjugated anti-CD45 (clone 30-F11, Invitrogen) and phycoerythrin–conjugated anti-CD11b (clone M1/70, Invitrogen) for 30 min under light-protected conditions at 4°C. After washing twice with cold staining buffer, labeled cells were analyzed and sorted using a BD FACSAria III flow cytometer (BD Biosciences) equipped with 488-nm and 561-nm lasers. Microglia were gated as CD11b^+^/CD45^low^ populations based on established immunophenotypic criteria (*62*, *63*). To validate purity, sorted cells were subjected to qRT-PCR analysis of microglial markers (*Tmem119*, *P2ry12*, and *Cx3cr1*) and immunofluorescence confirmation using Iba-1 (1:200; Wako) and CX3CR1 (1:100; BioLegend) antibodies post–cytospin preparation.

### Magnetic-activated cell sorting

Fresh brain tissues from 6-week-old C57BL/6J mice were rapidly immersed in precooled Hanks’ balanced salt solution (HBSS; Gibco) and transported on ice. Tissues were rinsed three times with ice-cold PBS (HyClone) to remove residual blood, and then enzymatically dissociated with 500 μl pf papain (20 U/ml; Worthington) and DNase I (10 μg/ml; Sigma-Aldrich) in HBSS at 37°C for 30 min under gentle agitation. The enzymatic reaction was terminated by centrifugation at 300*g* for 5 min at 4°C. The pellet was washed once with PBS and resuspended in 30% Percoll (GE Healthcare) diluted in HBSS for density gradient centrifugation (500*g* for 15 min at 18°C). The myelin-rich supernatant was carefully aspirated, and the cell pellet was reconstituted in ice-cold fluorescence-activated cell sorting (FACS) buffer [PBS with 2% fetal bovine serum (FBS) and 1 mM EDTA]. For microglial isolation, cells were incubated with CD11b MicroBeads (Miltenyi Biotec; 1:10 dilution) for 25 min at 4°C, washed twice with magnetic-activated cell sorting (MACS) buffer (Miltenyi Biotec), and then loaded onto a preequilibrated LS column mounted on a QuadroMACS Separator (Miltenyi Biotec). Unlabeled cells were removed by three washes with 3 ml of MACS buffer per wash. Bound CD11b-positive cells were eluted by column removal from the magnetic field, followed by forceful flushing with 5 ml of MACS buffer. Eluted microglia were pelleted (300*g* for 5 min) and subjected to purity validation through flow cytometry (>95% CD11b^+^/CD45^low^) and qRT-PCR analysis of microglial markers (*Tmem119*, *P2ry12*, and *Cx3cr1*), as detailed in previous section.

### Single-cell RNA sequencing

#### *Sample preparation*, *library construction, and reads filtering for scRNA-seq*

Retinas were harvested from P25 rd10 mice injected with AAV-*Cx3cr1-Tpm1*-EGFP or control AAV-*Cx3cr1*-EGFP (*n* = 8 to 10 mice per group) at P0 to P1. Dissociation of retinal tissues and isolation of microglia were performed as detailed in previous section. Microglia cell viability > 85% were prepared for scRNA-seq (*20*). The single-cell libraries were constructed with 10x Genomics platform, and the sequencing was performed using DNBSEQ sequencing platform (*20*). The sequencing depths of microglia in AAV-*Cx3cr1-Tpm1*-EGFP and AAV-*Cx3cr1*-EGFP groups were at 83,100 and 132,700 mean reads per cell, respectively, and the sequencing saturation rates of microglia in two groups were 89.42 and 92.99%, respectively. Sample demultiplexing, barcode processing, and single-cell counting were performed using the Cell Ranger (version 5.0.1). RNA reads were aligned with the mouse reference genome (refdata-gex-mm10-2020-A) using STAR alignment before calculating unique molecular identifier (UMI) counts (*20*). The raw output data were processed and analyzed using the Seurat package (version 5.0.1) in R software (version 4.3.1). The quality control was performed by filtering out cells identified with (i) a gene count less than 200, (ii) a gene count greater than the maximum gene count * 90%, and (iii) the top 15% of cells with the highest proportion of mitochondria reads. The cell cycle influences were corrected by Seurat package, and removal of potential doublets were conducted by DoubletFinder package (version 2.0.2) (*20*). A total of 2347 and 1410 microglia from AAV-*Cx3cr1-Tpm1*-EGFP and AAV-*Cx3cr1*-EGFP groups was used for further analysis, respectively.

#### Dimensionality reduction and cell clustering

After normalizing the filtered gene-barcode matrices with the “NormalizedData” function in R using Seurat package, the top 2000 highly variable genes were identified with “FindVariableFeatures” function. Furthermore, gene expression matrices were scaled and centered using “ScaleData” function, followed by performing principal components analysis and uniform manifold approximation and projection (UMAP) dimension reduction using the top 20 principal components (PCs). To remove the batch effects across all samples, all data were processed using “FindIntergrationAnchors” and “IntegrateData” functions in Seurat with default parameters (*20*). Clustering analysis was performed with “FindNeighbors” (first 20 PCs) and “FindClusters” (resolution = 0.4) functions, which ultimately yielded seven cell clusters. The cell annotation was conducted on the basis of the top marker gene expressions in each cluster, which were calculated by the “FindAllMarkers” function (*20*).

#### *DEG identification*, *pathway enrichment analysis*, *GSEA*, *and UCell analysis*

DEGs were identified using Seurat’s FindAllMarkers/FindMarkers functions with Wilcoxon rank sum test under the following thresholds: (i) minimum detection fraction: min.pct = 0.1 in either group, (ii) log–fold change threshold: logfc.threshold = 0.25, (iii) significance cutoff: Benjamini-Hochberg adjusted *P* < 0.05 (*20*). The cluster-specific DEGs were used for GO and KEGG pathway enrichment analysis using ShinyGO0.77 (http://bioinformatics.sdstate.edu/go/). The protein-protein interaction among DEGs was analyzed using STRING (version 12.0) and Cytoscape (version 3.10.1) (*64*). To identify pathways, which were induced or repressed in the cell clusters, we performed GSEA using gene sets from KEGG database (*65*). GSEA analysis in specific cell cluster was conducted using the “GSEA” and “gseaplot2” functions from the GSEABase (version 1.64.0), enrichplot (version 1.22.0), and clusterProfiler (version 4.10.0) packages in R software. Cell-level pathway activity scoring was performed using UCell (version 2.7.6) to calculate the activity level of pathways in each individual cell and visualize the activity scores on the UMAP.

#### Analysis of cell-cell interactions

Cell-cell communications were performed with CellChat (version 1.6.1) (*66*). CellChat objects were generated from the Seurat object, and the data were preprocessed using the “subsetData,” “identifyOverExpressedGenes,” and “identifyOverExpressedInterations” functions with default parameters. The “computeCommunProb” function was then used to calculate communication probability and generate a communication network. The analysis was performed using the CellChatDB mouse database. To identify the cell-cell interaction among different microglia clusters, we used “netVisual_chord_gene” and “netVisual_individual” functions (*20*). The identification of dominant senders, receivers, mediators, and influencers in the intercellular communication network was calculated and visualized by “netAnalysis_computeCentrality” and “netAnalysis_signalingRole_network” function, respectively (*67*).

#### TF module analysis

We used the SCENIC package (version 1.3.1) in R software to infer TFs and gene regulatory networks for microglial clusters (*68*). The RcisTarget databases “mm9-500 bp-upstream-7species.mc9nr.feather” and “mm9-tss-centered-10 kb-7species.mc9nr.feather,” containing TF motif scores for gene promoters and around transcription start sites for the mouse reference genome, were used for reference. We used “runCorrelation” and “GENIE3” to calculate the correlation of candidate regulators. The downstream pipeline for constructing gene regulatory networks included “runSCENIC_1_coexNetwork2modules,” “runSCENIC_2_createRegulons,” “runSCENIC_3_scoreCells,” and “runSCENIC_4_aucell_binarize.” TF genes with high regulatory activity and expression in this data were shown by heatmap plot (R package) (*20*, *67*).

#### Signed consensus weighted gene coexpression network analysis

scWGCNA was performed as previously described (*69*). All *Tpm1*-expressing microglia were used for metacell computation in SEACells v1.4. The coexpression network was constructed following the established framework (*69*, *70*) with modifications for single-cell data. Briefly, adjacency matrix was calculated using biweight midcorrelation with signed network preservation, and optimal power value (β = 12) was determined by scale-free topology criterion (*R*^2^ > 0.85) via the pickSoftThreshold function, and topological overlap matrix (TOM) was generated with TOMsimilarityFromExpr using β = 12 and TOMType = “signed.” Modules were detected through hierarchical clustering (average linkage clustering of 1-TOM dissimilarity matrix) and dynamic tree cutting [minClusterSize = 100, deepSplit = 4, and MEDissThres = 0.2 (1 – Pearson correlation)] and eigengene calculation (first principal component of module expression profiles via moduleEigengenes). Eigengenes were correlated with microglial activation states using Pearson correlation (continuous traits), linear regression adjusted for technical covariates (sequencing depth, mitochondrial%), and Benjamini-Hochberg false discovery rate (FDR) correction for multiple comparisons. Hub genes were defined using intramodular connectivity (kME) parameters of the WGCNA package. GSEA was done using EnrichR.

### In vitro experiments

#### Cell lines

The murine microglial cell line BV2 was kindly provided by H. Xu (Southwest Eye Hospital, Chongqing, China) (*71*), and the mouse photoreceptor-derived 661W cell line was generously gifted by M. Al-Ubaidi (University of Oklahoma) (*72*). Both cell lines were maintained in Dulbecco’s modified Eagle’s medium (DMEM; Gibco) supplemented with 10% heat-inactivated FBS (Gibco), 1% penicillin-streptomycin (10,000 U/ml; HyClone), and 2 mM GlutaMAX (Thermo Fisher Scientific) at 37°C in a humidified 5% CO_2_ atmosphere. Cells were passaged every 3 to 4 days at 80 to 90% confluence using 0.25% trypsin-EDTA (Gibco) and subcultured in T75 flasks (Corning).

#### Plasmids and siRNA transfection

The *Tpm1* overexpression plasmid (*Tpm1* plasmid) and empty vector control (control plasmid) were obtained from Ubigene Biotechnology (Guangzhou, China) (*16*). siRNA targeting *Tpm1*, *Cd45*, *Lgals9*, *Apoe*, *Fabp5*, *Mapk6*, and scrambled siCTR was synthesized by Thermo Fisher Scientific (Hong Kong) and Synbio Technologies (Suzhou, China) with the following sequences (table S1). Primary microglia were isolated from 6-week-old male C57BL/6J mice as previously described. Cells were seeded in 12-well plates (1 × 10^5^ cells per well) and precultured for 24 hours in DMEM (Gibco) containing 10% FBS (Gibco), 1% penicillin-streptomycin (10,000 U/ml; HyClone), and 2 mM GlutaMAX (Thermo Fisher Scientific) at 37°C/5% CO_2_. Following medium renewal, cells underwent continuous culture for 6 days to achieve >90% confluence before transfection. For transfection, 2 μg of plasmid DNA or 100 pmol of siRNA was mixed with 3 μl of Lipofectamine 3000 (Invitrogen) or 6 μl of Lipofectamine RNAiMAX Reagent in Opti-MEM (Gibco) according to the manufacturer’s protocol. The DNA-lipid complexes were added to cells for 6 to 8 hours, after which the transfection medium was replaced with fresh complete medium. Cells were harvested 36 hours posttransfection for downstream analyses. Transfection efficiency was validated by qRT-PCR quantification.

#### LPS stimulation of BV2 microglia

BV2 cells were seeded into 12-well plates at a density of 1 × 10^5^ cells per well and cultured for 24 hours in complete DMEM (10% FBS and 1% penicillin-streptomycin) to reach 70 to 80% confluence. Cells were then transfected with si*Tpm1* or scrambled siCTR using Lipofectamine RNAiMAX (Invitrogen) as previously described. Following 30 hours of transfection, cells were stimulated with ultrapure LPS (1 μg/ml; *Escherichia coli* O111:B4; Sigma-Aldrich) diluted in serum-free DMEM for 6 hours at 37°C in 5% CO_2_. Poststimulation, cells were rinsed twice with PBS and harvested for RNA extraction (TRIzol, Invitrogen) and qRT-PCR analysis of inflammatory markers (*Tpm1*, *Il1b*, *Il6*, *Tnf*α, and *Nos2*).

#### Cell viability assay by Cell Counting Kit-8

BV2 cells were seeded in 96-well plates at 5000 cells per well and transfected with si*Tpm1* or scrambled siCTR, followed by LPS stimulation as outlined above. After treatment, cells were gently washed twice with PBS (pH 7.4) and incubated with 10 μl of Cell Counting Kit-8 (CCK-8) reagent (HY-K0301, MCE) diluted in 90 μl of serum-free DMEM for 1 hour at 37°C in 5% CO_2_. Absorbance was measured at 450 nm with a reference wavelength of 600 nm using a microplate reader. Three independent biological replicates were performed, each containing five technical replicates.

#### Wound healing assay for microglial migration

BV2 cells were plated in six-well plates at 1.5 × 10^5^ cells per well and transfected with si*Tpm1* or scrambled siCTR using Lipofectamine RNAiMAX (Invitrogen), followed by LPS (1 μg/ml; *E. coli* O111:B4; Sigma-Aldrich) stimulation as previously described. Upon reaching 95% confluence, cross-shaped scratches in each well were generated using a 200-μl sterile pipette tip (Corning). Detached cells were removed by three gentle washes with PBS, and fresh serum-free DMEM containing 1% penicillin-streptomycin was added. Plates were incubated at 37°C in 5% CO_2_, with phase-contrast images captured at 0, 12, and 24 hours postwounding using an Olympus IX83 microscope equipped with a 10× objective. Wound closure was quantified by measuring residual wound area (%) using the ImageJ v1.53 software (National Institutes of Health) with the following equation: closure rate = (1 − *A_t_*/*A*_0_) × 100, where *A*_0_ = initial wound area (0 hours) and *A_t_* = wound area at time *t*. Three independent experiments were performed.

#### MK2-IN-3 treatment of BV2 microglia

BV2 cells were seeded in 12-well plates at a density of 0.5 × 10^5^ cells per well. Cells were transfected with *Tpm1* plasmid or scrambled control plasmid using Lipofectamine 3000 (Invitrogen) according to the manufacturer’s protocol, with plasmids diluted in Opti-MEM (Gibco). At 30 hours posttransfection, cells were treated with 5 μM MK2-IN-3 (MCE) for 6 hours, followed by cell collection for downstream analysis.

#### Detection of cellular lipid and free cholesterol in microglia

Primary microglia isolated from 6-week-old C57BL/6J mice were transfected with the *Tpm1* plasmid or an empty vector control, followed by transfection with si*Apoe* or si*Fabp5* as described in the previous section. Thirty-six hours posttransfection, cells were fixed with 4% PFA for 15 min. Nile Red, BODIPY C11, and Filipin staining were performed according to established protocols.

#### Isolation of retinal cells or rod photoreceptors

Retinal tissues from P25 rd10 or C57BL/6J mice were dissociated into single-cell suspensions using the enzymatic protocol described in previous section. Cell suspensions were centrifuged at 300*g* for 5 min at 4°C, washed once with PBS (pH 7.4), and resuspended in ice-cold FACS buffer (PBS with 2% FBS and 1 mM EDTA). For rod photoreceptor isolation, single-cell suspensions from P25 C57BL/6J retinas were filtered through a 40-μm cell strainer (Corning) and sorted using a BD FACSAria III flow cytometer (BD Biosciences) equipped with an 80-μm nozzle. Initial gating was performed on the basis of forward scatter (FSC-A) and side scatter (SSC-A) parameters to exclude debris and cell aggregates. A distinct population with low FSC/SSC profiles, corresponding to rod photoreceptors (*73*), was collected at a flow rate of ≤1500 events/s. Sorted cells were validated for purity through immunofluorescence detection: >95% positivity for rod-specific markers (Rhodopsin; 1:500; ab3267, Abcam).

#### Cell supernatant stimulation in rod/cone photoreceptors

Following transfection with *Tpm1* plasmid or control plasmid in primary microglia isolated from 6-week-old C57BL/6J mouse brain, cell supernatants from *Tpm1* plasmid–treated (MCM2) or control plasmid–treated microglia (MCM1) were collected. Isolated rod photoreceptors from P25 C57BL/6J retinas or 661W cone photoreceptors were seeded in a 12-well plate and incubated with MCM1 and MCM2 for 12 hours, followed by quantification analysis of apoptosis signatures (*Bax*, *Bcl2*, and *Caspase-3*).

#### MCM treatment in photoreceptors

Primary microglia isolated from 6-week-old C57BL/6J mice were transfected with *Tpm1* plasmid or empty vector control as described in previous section. Conditioned media were collected 36 hours posttransfection, centrifuged at 2000*g* for 10 min to remove cellular debris, and designated as MCM2 (*Tpm1* plasmid treated) or MCM1 (control plasmid treated). Freshly isolated rod photoreceptors from P25 C57BL/6J retinas described above or 661W cone photoreceptor cells were plated in 12-well culture inserts (1 × 10^5^ cells per well; Corning Transwell) and exposed to MCM1 or MCM2 for 12 hours. Apoptotic responses were assessed through qRT-PCR analysis of apoptosis signatures (*Bax*, *Bcl2*, and *Caspase-3*).

#### Coculture of microglia and retinal cells

Primary microglia isolated from 6-week-old C57BL/6J mice were seeded in 12-well plate (1 × 10^5^ cells per well) and transfected with *Tpm1* plasmid or empty vector control as detailed in previous section. At 36 hours posttransfection, microglia were cocultured with freshly isolated retinal cells from P25 rd10 mice for 12 hours at 37°C. Phagocytic activity was quantified with immunofluorescence colocalization analysis.

#### Immunoblotting

BV2 cells were transfected with si*Tpm1* or scrambled siCTR using Lipofectamine RNAiMAX (Invitrogen) as previously described. At 30 hours posttransfection, cells were stimulated with ultrapure LPS (1 μg/ml; *E. coli* O111:B4; Sigma-Aldrich) diluted in serum-free DMEM for 6 hours at 37°C/5% CO_2_. Poststimulation, cells were washed twice with ice-cold PBS and lysed in RIPA buffer (Abcam) containing 1× protease/phosphatase inhibitor cocktail (Roche). Lysates were centrifuged at 12,000*g* for 15 min at 4°C, and supernatant protein concentrations were quantified via BCA assay kit (Thermo Fisher Scientific). Western blot analysis was performed as described in previous section.

#### Quantitative real-time PCR

After different treatments, BV2 cells or primary microglia isolated from 6-week-old C57BL/6J mice were collected, and total RNA were extracted with TransZol Up Plus reagent (TransGen Biotech) or TRIzol Reagent (Thermo Fisher Scientific). cDNA synthesis and qPCR quantification were performed as previously detailed. Primer sequences were listed in table S1.

### Statistical analysis

All experiments included three independent biological replicates with experimental groups randomized using permuted block design. Sample size was specified in corresponding figure legends. Data plotting and statistical tests were performed using the GraphPad Prism software version 8.0. Data are represented as means ± SEMs and analyzed with unpaired two-tailed Student’s *t* test or one-way and two-way analysis of variance (ANOVA) with Tukey’s or Šídák’s multiple-comparison test. In all graphs, statistical significance was described as *P < 0.05, **P < 0.01, ***P < 0.001, and ****P < 0.0001.
