# Supplementary material for: TPM1 drives cytoskeleton-immunometabolism coupling and LGALS9/CD45-mediated neuroinflammatory propagation in retinitis pigmentosa
Source: Sci Adv. 2026 May 27;12(22):eaea6467. doi: 10.1126/sciadv.aea6467 (PMC13215182; doi:10.1126/sciadv.aea6467)
Supplement: Supplementary file 1 — Figs. S1 to S10 Table S1 [file sciadv.aea6467_sm.pdf]

Science Advances
AAAS

Supplementary Materials for

TPM1 drives cytoskeleton-immunometabolism coupling and LGALS9/CD45-mediated neuroinflammatory propagation in retinitis pigmentosa

Rong Li *et al.*

Corresponding author: Rong Li, 18073861r@connect.polyu.hk; Bin Lin, b.lin@polyu.edu.hk

Sci. Adv. **12**, eaea6467 (2026)
DOI: 10.1126/sciadv.aea6467

This PDF file includes:

Figs. S1 to S10
Table S1

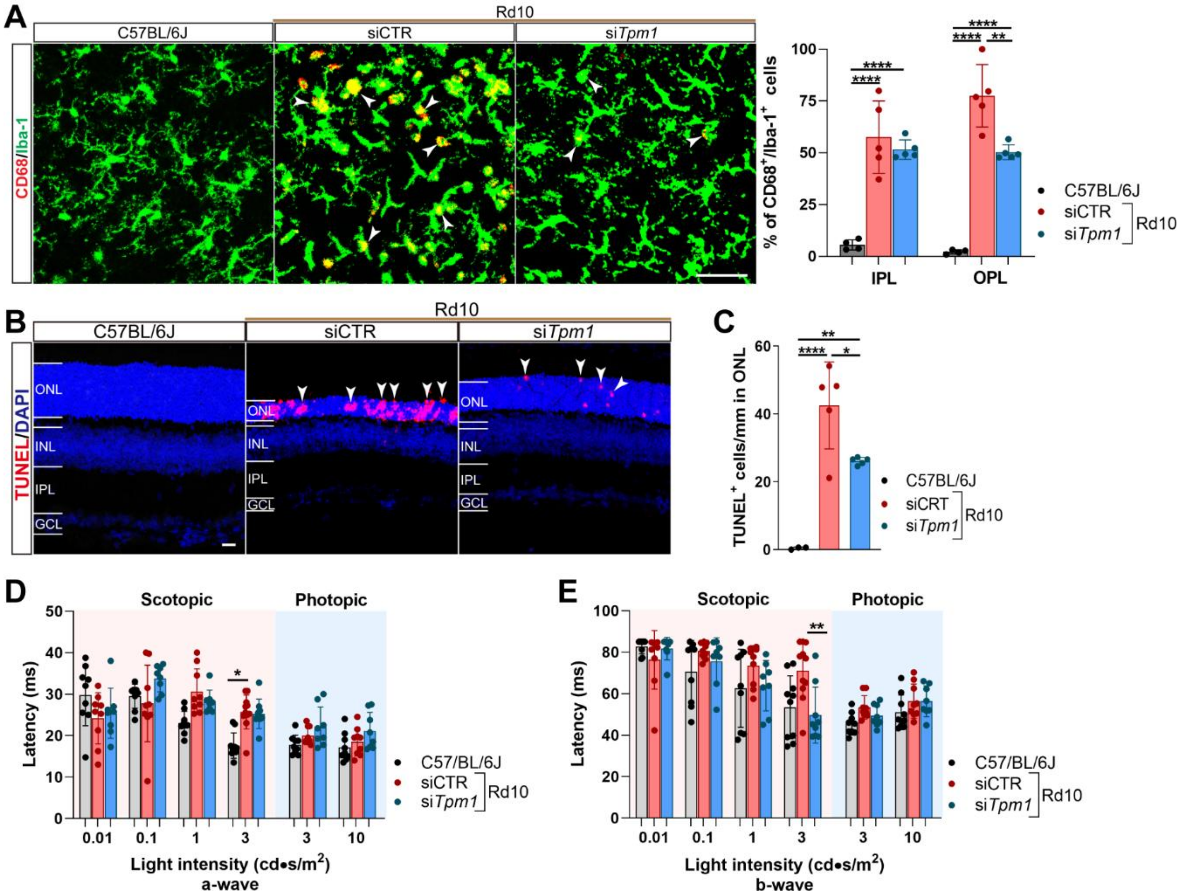

fig. S1. *Tpm1* knockdown mitigates microglial activation and photoreceptor apoptosis in the retinas of rd10 mice. (A) Retinal wholemounts stained with CD68 and Iba-1 antibodies and quantification of CD68$^+$ microglia in both the OPL and IPL of the retinas from P25 rd10 mice after intravitreal injection with si*Tpm1* or siCTR and age-matched C57BL/6J mice (n=4-5 mice/group). The white arrowheads show CD68$^+$ microglia. Four sampling areas with 638.9 μm x 638.9 μm squares along the dorsal-ventral axis of retinal whole-mounts at 200 μm and 1 mm from the optic nerve head on both sides were photographed. Scale bar, 20 μm. **(B)** TUNEL staining in retinal sections. The white arrowheads show TUNEL$^+$ cells in the ONL. Three views in each retinal section at 100 μm (central), 1 mm (middle) and 1.8 mm (peripheral) from the optic nerve head along the dorsal and ventral directions were captured. Scale bar, 20 μm. **(C)** quantification of TUNEL$^+$ cells in the ONL of the retinas (n=5 mice/group). **(D-E)** Quantification of a- (**D**) and b-wave (**E**) latencies in both scotopic and photopic conditions in P25 rd10 mice after intravitreal injection with si*Tpm1* or siCTR and age-matched C57BL/6J mice (n=8-9 mice/group). The data are presented as the means ± SEMs and were analysed via one-way ANOVA (**C**) or two-way ANOVA (**A, D-E**) with Tukey's multiple comparison test ($*P < 0.05$, $**P < 0.01$, $****P < 0.0001$).

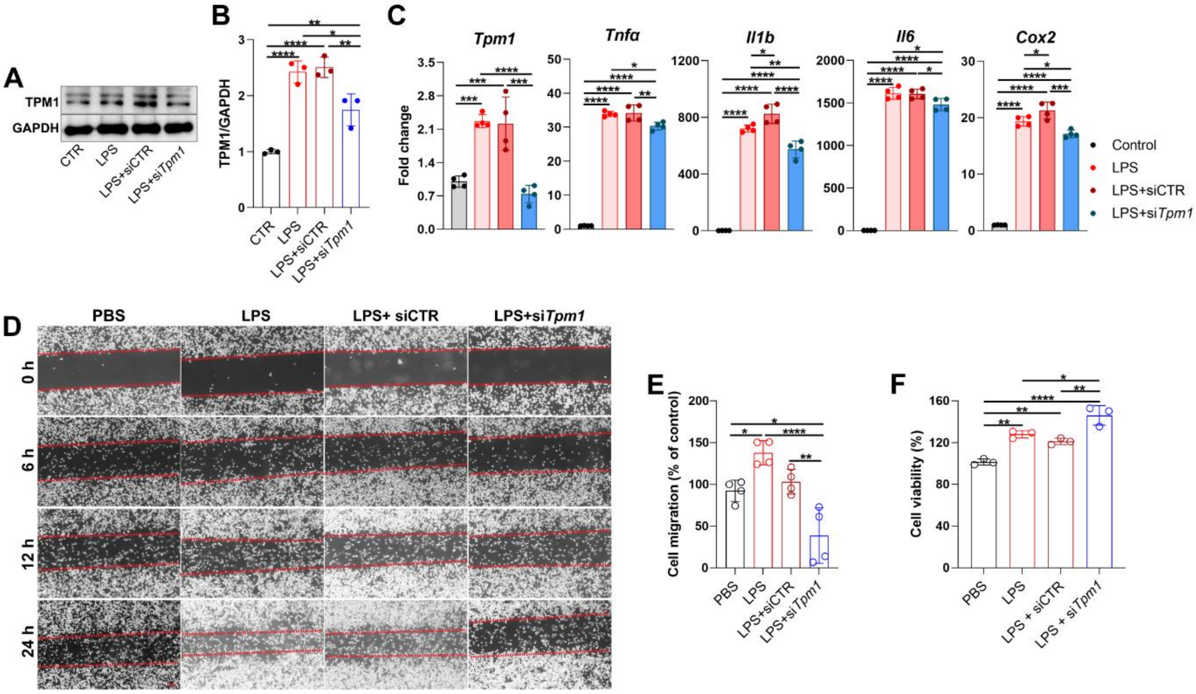

fig. S2. *Tpm1* silencing attenuates LPS-induced BV2 microglial activation. (A-B) Western blot (**A**) and quantification of TPM1 expression (**B**) in BV2 cells after treatment with si*Tpm1* or siCTR followed by stimulation with LPS. **(C)** qPCR analysis of *Tpm1* and proinflammatory cytokines expression in BV2 cells. **(D-E)** The time-lapse microscopy images of wound closure (**D**) and quantification of cell migration (**E**) in BV2 cells after treatment with si*Tpm1* or siCTR followed by stimulation with LPS. The red dotted lines define the area lacking cells. Scale bar, 100 μm. **(F)** Quantification of cell viability of BV2 cells after treatment with si*Tpm1* or siCTR followed by stimulation with LPS. The results shown represent 3-4 independent experiments. The data are presented as the means ± SEMs and were analysed via one-way ANOVA with Tukey's multiple comparison test (*$P < 0.05$, **$P < 0.01$, ***$P < 0.001$, ****$P < 0.0001$).

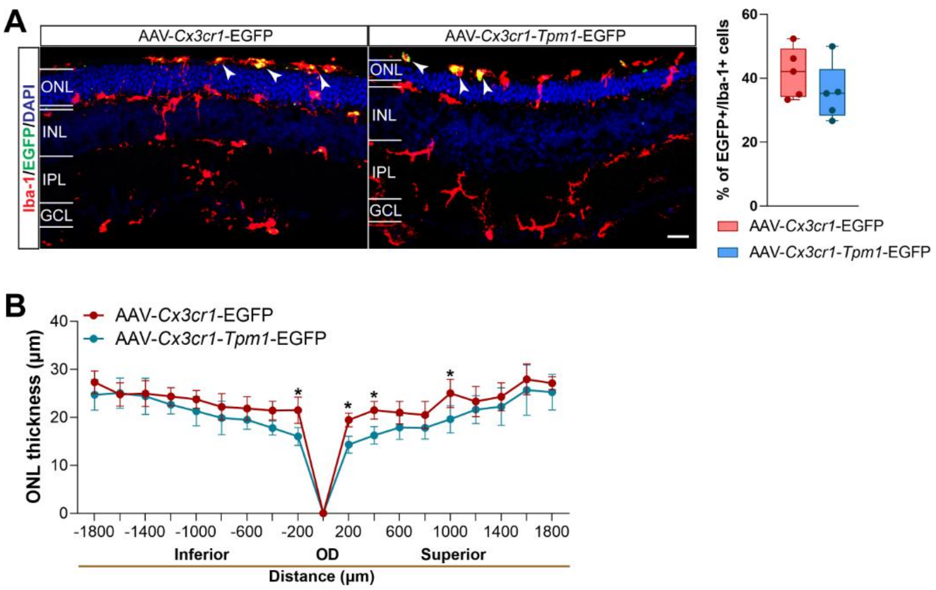

fig. S3. Microglia-specific AAV transduction efficiency. (A) Retinal sections stained with Iba-1 antibody and quantification of $EGFP^+$ microglia in the retinas of P25 rd10 mice after subretinal injection with AAV-*Cx3cr1*-*Tpm1*-EGFP or AAV-*Cx3cr1*-EGFP (n=5 mice/group). The white arrowheads show $EGFP^+$ microglia. Scale bar, 20 µm. Three views in each retinal section at 100 µm (central), 1 mm (middle) and 1.8 mm (peripheral) from the optic nerve head along the dorsal and ventral directions were captured. **(B)** Spider plots showing ONL thickness measured at distances of 200, 400, 600, 800, 1000, 1200, 1400, 1600, and 1800 µm from the optic disc (OD) toward the superior or inferior retinal tip (n = 5 mice/group). Data are presented as mean ± SEMs and were analyzed using unpaired two-tailed Student's *t*-tests (**A**) or two-way ANOVA followed by Šídák's multiple comparisons test (**B**) (*P < 0.05, **P < 0.01).

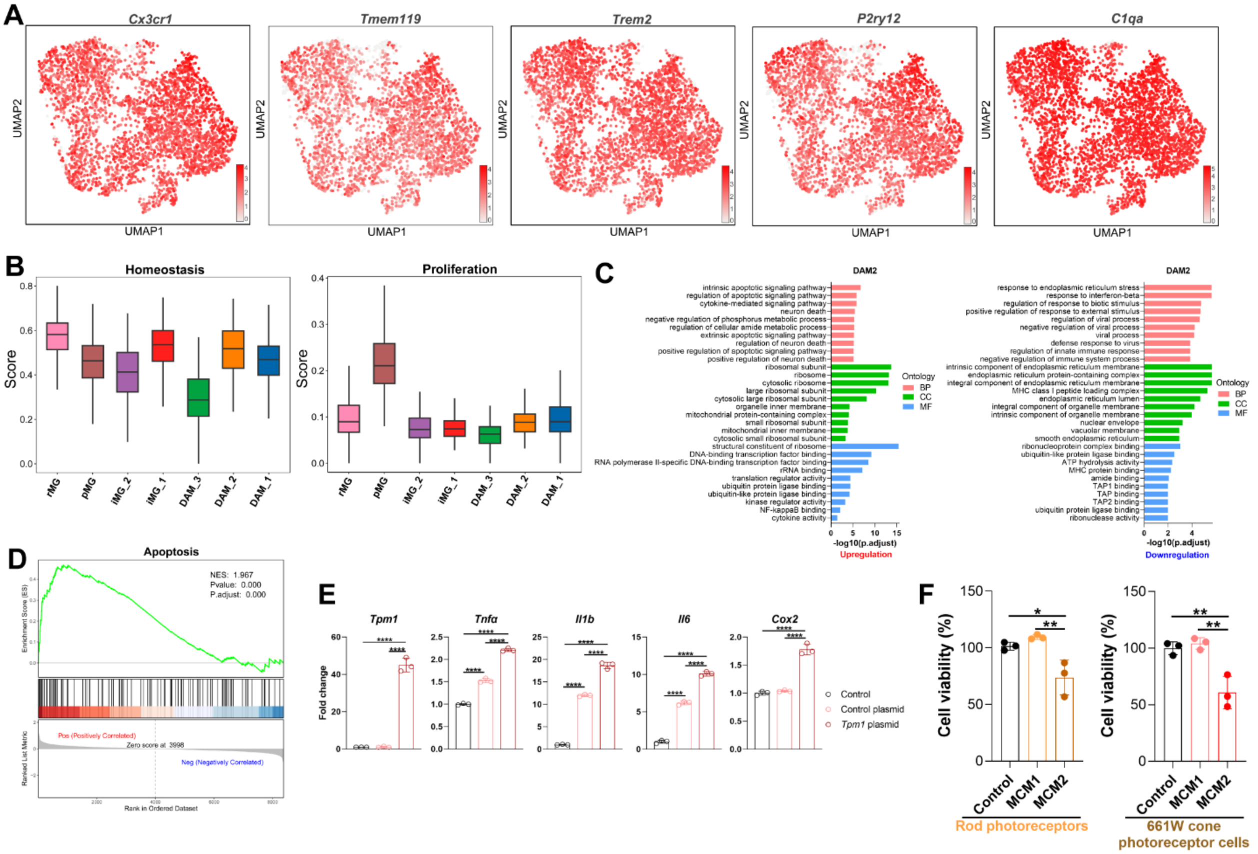

fig. S4. Single-cell transcriptomic profiling of microglia in the retinas of rd10 mice following AAV-*Cx3cr1-Tpm1*-EGFP treatment. (A) UMAP plots showing *Cx3cr1, Tmem119, Trem2, P2ry12* and *C1qa* expression across all different microglial clusters from the retinas of P25 rd10 mice treated with AAV-*Cx3cr1-Tpm1*-EGFP or AAV-*Cx3cr1*-EGFP. **(B)** Box plots showing the UCell score of homeostasis and proliferation pathways in different clusters. **(C)** Gene ontology (GO) enrichment analysis in biological process (BP), cellular components (CC) and molecular function (MF) of DEGs in cluster DAM2. **(D)** GSEA analysis of apoptosis pathway in DAM2 cluster. **(E)** qPCR analysis of *Tpm1* and proinflammatory cytokines expression in microglia isolated from 6-week-old C57BL/6J mice and transfected with *Tpm1* plasmid or control plasmid. **(F)** Quantification of cell viability of rod photoreceptors isolated from P25 C57BL/6J retinas and of 661W cone photoreceptors after treatment with MCM1 and MCM2. The results shown represent 3 independent experiments. The data are presented as the means ± SEMs and were analysed via one-way ANOVA with Tukey's multiple comparison test (**E, F**) (*$P < 0.05$, **$P < 0.01$, ****$P < 0.0001$).

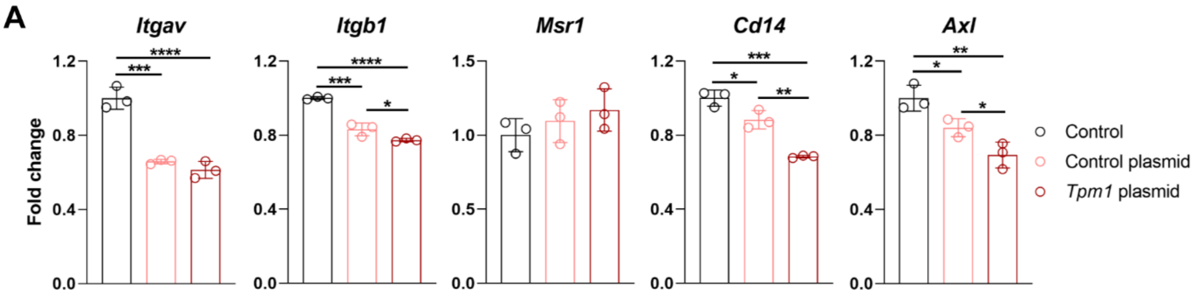

fig. S5. Impaired phagocytosis in *Tpm1*-overexpressing microglia. (A) qPCR analysis of phagocytosis-associated genes including *Itgav, Itgb1, Msr1, Cd14* and *Axl* in microglia isolated from 6-week-old C57BL/6J mice and transfected with *Tpm1* plasmid or control plasmid. The results shown represent 3 independent experiments. The data are presented as the means ± SEMs and were analysed via one-way ANOVA with Tukey's multiple comparison test (*$P < 0.05$, **$P < 0.01$, ***$P < 0.001$, ****$P < 0.0001$).

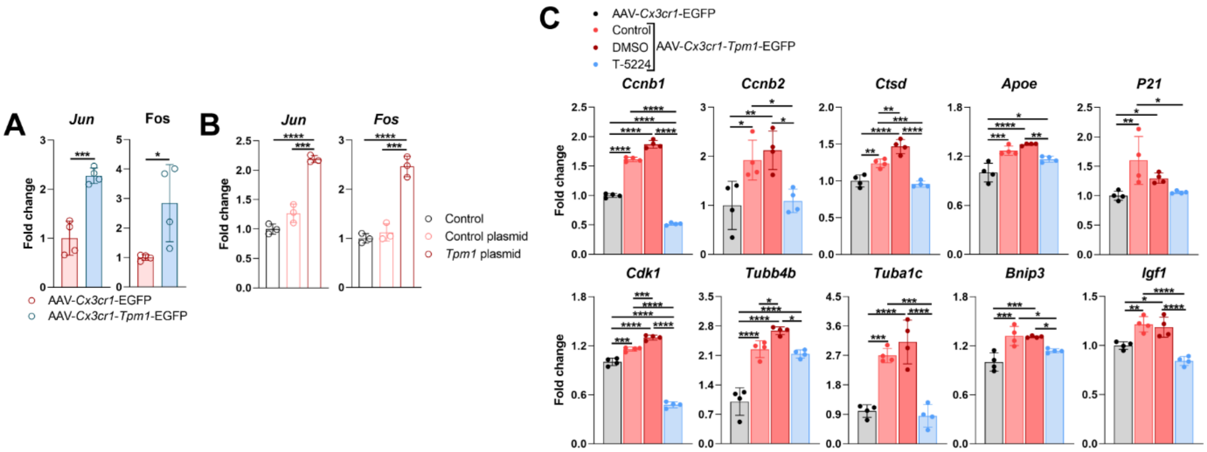

fig. S6. AP-1 signaling mediates *Tpm1*-induced senescence in the retinas of rd10 mice. (A) qPCR analysis of *Jun* and *Fos* expression in P25 rd10 retinas treated with AAV-*Cx3cr1-Tpm1*-EGFP or AAV-*Cx3cr1*-EGFP (n=4 mice/group). **(B)** qPCR analysis of *Jun* and *Fos* in microglia isolated from 6-week-old C57BL/6J mice and transfected with *Tpm1* plasmid or control plasmid. The results shown represent 3 independent experiments. **(C)** qPCR analysis of expression of senescence-associated genes including *Ccnb1, Ccnb2, Ctsd, Apoe, P21, Cdk1, Tubb4b, Tuba1c, Bnip3* and *Igf1* in P25 rd10 retinas treated with AAV-*Cx3cr1-Tpm1*-EGFP or AAV-*Cx3cr1*-EGFP followed by intravitreal injection with T-5224 or DMSO (n=4 mice/group). The data are presented as the means ± SEMs and were analysed via unpaired two-tailed Student's *t* tests (**A**) or one-way ANOVA with Tukey's multiple comparison test (**B-C**) (*$P < 0.05$, **$P < 0.01$, ***$P < 0.001$, ****$P < 0.0001$).

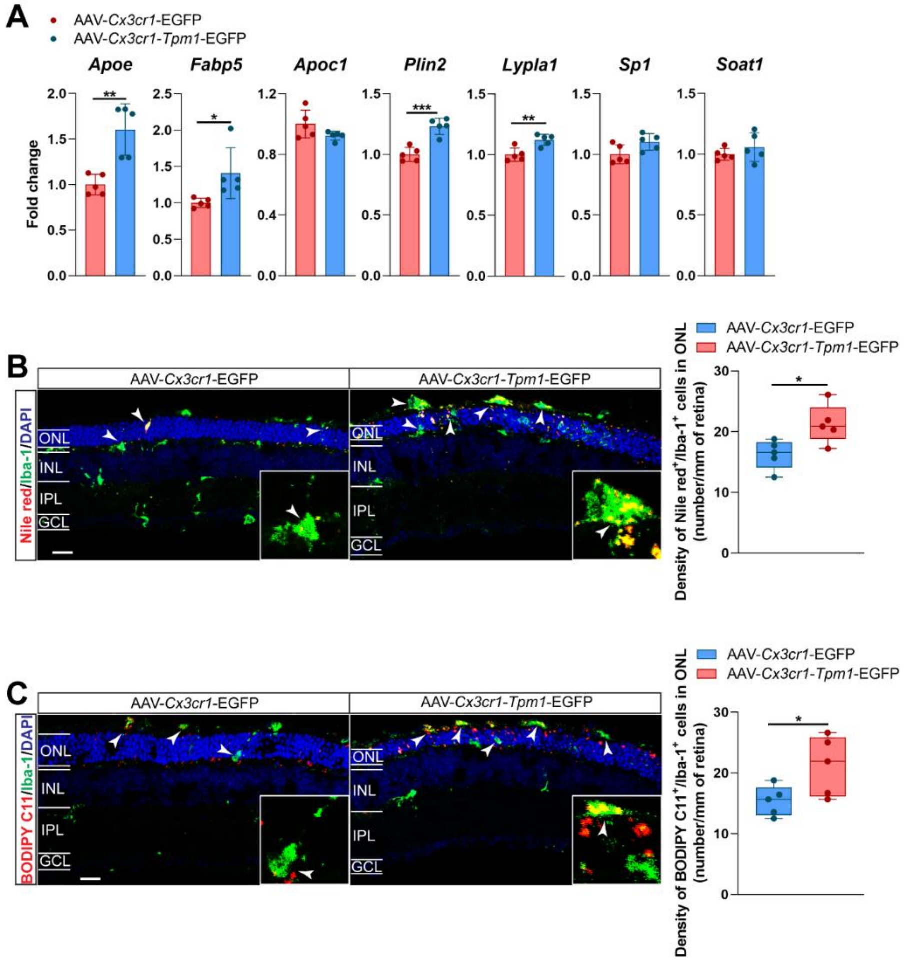

fig. S7. *Tpm1* regulates *Apoe/Fabp5*-driven lipid dysregulation in microglia in RP. (A) qPCR validation of *Tpm1* and lipid metabolism regulators (*Apoe/Fabp5/Apoc1/Plin2/Lypla1/Sp1/Soat1*) in the retinas of rd10 mice administrated with AAV-*Cx3cr1-Tpm1*-EGFP or control vector (n=5 mice/group). **(B-C)** Representative confocal images showing lipid accumulation (Nile red: lipid droplets; BODIPY C11: neutral lipids) in microglia of the retinas from rd10 mice administrated with AAV-*Cx3cr1-Tpm1*-EGFP or control vector (n=5 mice/group). Yellow arrowheads indicate Nile red$^+$ or BODIPY C11$^+$ microglia. Scale bars: 20 μm. Density of Nile red$^+$ or BODIPY C11$^+$ microglia in ONL was quantified. For image acquiring (**B,C**), three views in each retinal section at 100 μm (central), 1 mm (middle) and 1.8 mm (peripheral) from the optic nerve head along the dorsal and ventral directions were captured. The data are presented as the means ± SEMs and were analysed via unpaired two-tailed Student's *t* tests (*$P < 0.05$, **$P < 0.01$, ***$P < 0.001$).

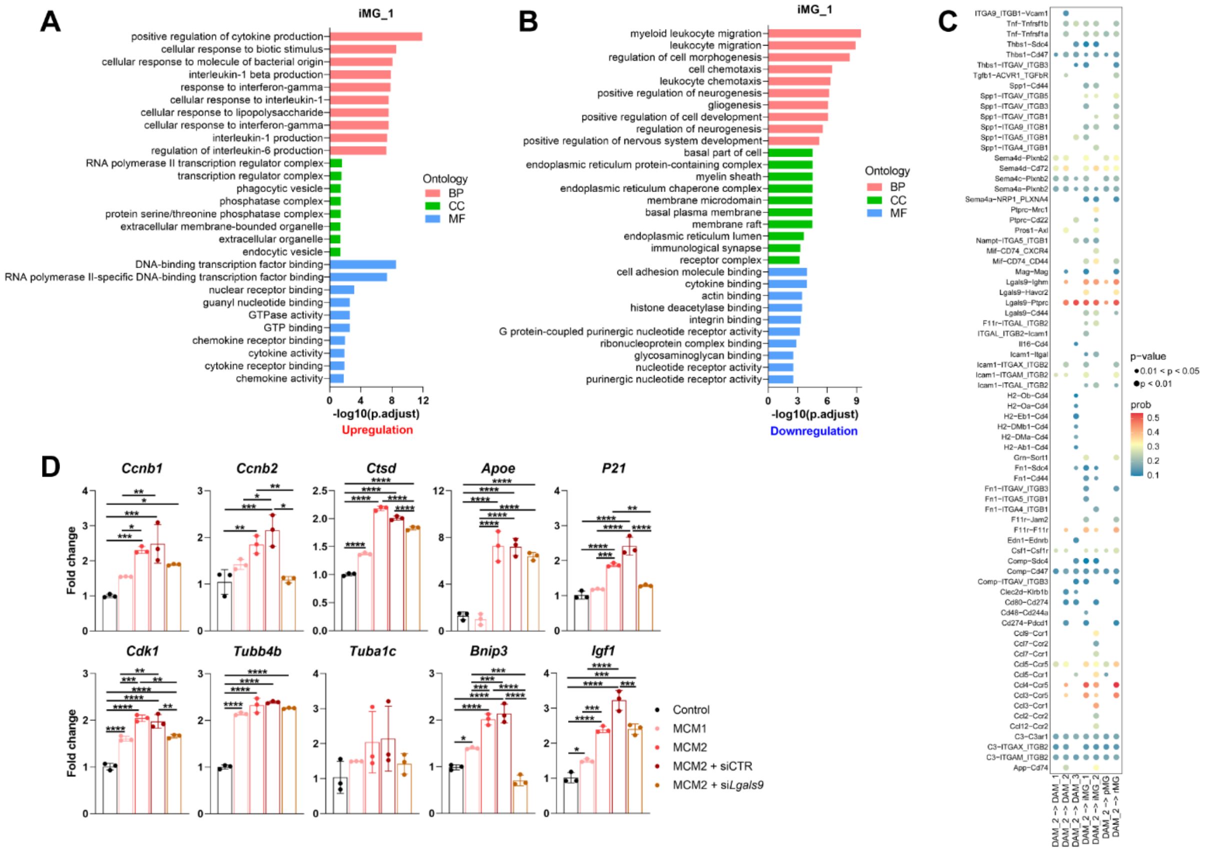

fig. S8. LGALS9/CD45 axis regulates intercellular communication between microglia following microglial *Tpm1* overexpression in the retinas of rd10 mice. (A-B) Gene ontology (GO) enrichment analysis in biological process (BP), cellular components (CC) and molecular function (MF) of DEGs in iMG_1 cluster. **(C)** The significant L-R pairs between DAM2 and other microglial clusters. **(D)** qPCR analysis of expression of senescence-associated genes including *Ccnb1, Ccnb2, Ctsd, Apoe, P21, Cdk1, Tubb4b, Tuba1c, Bnip3* and *Igf1* in microglia isolated from 6-week-old C57BL/6J mice and transfected with si*Lgals9* or siCTR followed by treatment with MCM1 or MCM2. The results shown represent 3 independent experiments. The data are presented as the means ± SEMs and were analysed via one-way ANOVA with Tukey's multiple comparison test (**D**) (*$P < 0.05$, **$P < 0.01$, ***$P < 0.001$, ****$P < 0.0001$).

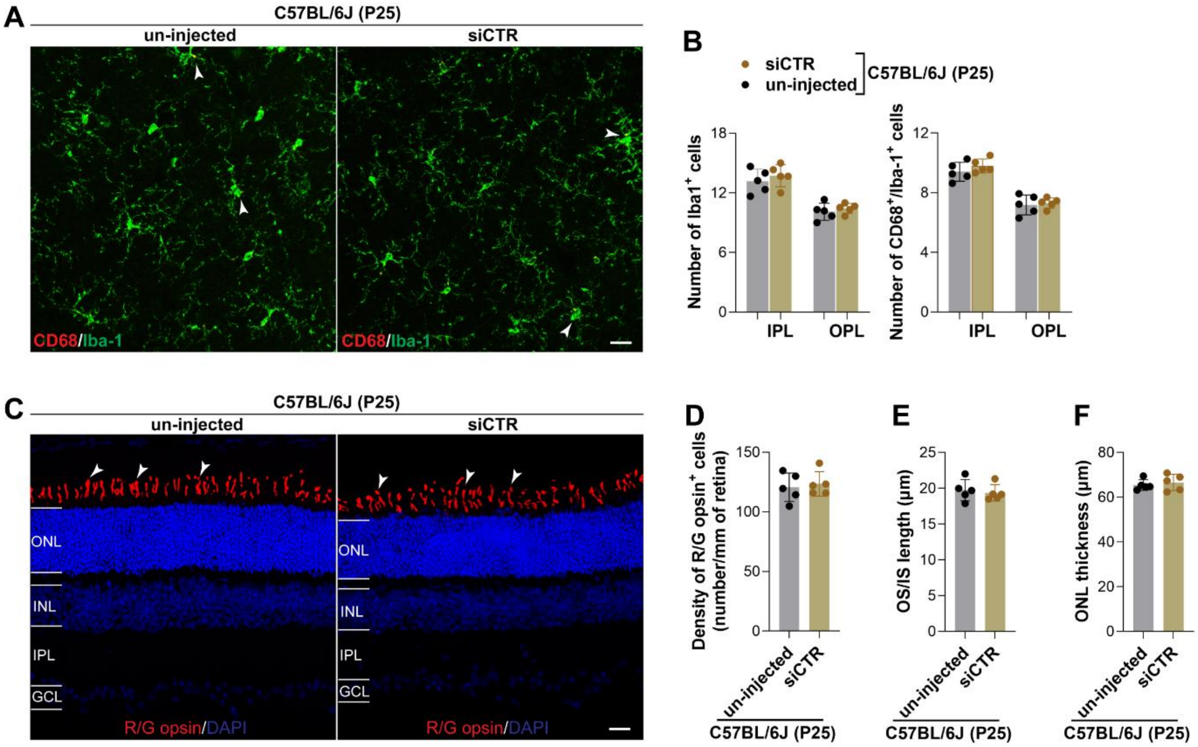

fig. S9. Intravitreal injection of siCTR does not induce microgliosis or photoreceptor degeneration in C57BL/6J mice. (A–B) Retinal wholemounts immunostained for Iba-1 and CD68 (**A**), with quantification of Iba-1^{+} and CD68^{+}/Iba-1^{+} cells in the IPL and OPL (**B**) from P25 C57BL/6J mice with or without siCTR injection (n = 5 mice/group). White arrowheads indicate Iba-1^{+}/CD68^{+} cells. Four sampling areas with 638.9 µm x 638.9 µm squares along the dorsal-ventral axis of retinal whole-mounts at 200 µm and 1 mm from the optic nerve head on both sides were photographed. **(C–F)** Retinal sections labeled with R/G opsin (**C**), and quantification of R/G opsin^{+} cell density (**D**), OS/IS length (**E**), and ONL thickness (**F**) in P25 C57BL/6J mice with or without siCTR injection (n = 5 mice/group). Three views in each retinal section at 100 µm (central), 1 mm (middle) and 1.8 mm (peripheral) from the optic nerve head along the dorsal and ventral directions were captured. White arrowheads mark R/G opsin^{+} cells. Scale bar: 20 µm (**A, C**). The data are presented as the means ± SEMs and were analysed via unpaired two-tailed Student's *t* tests.

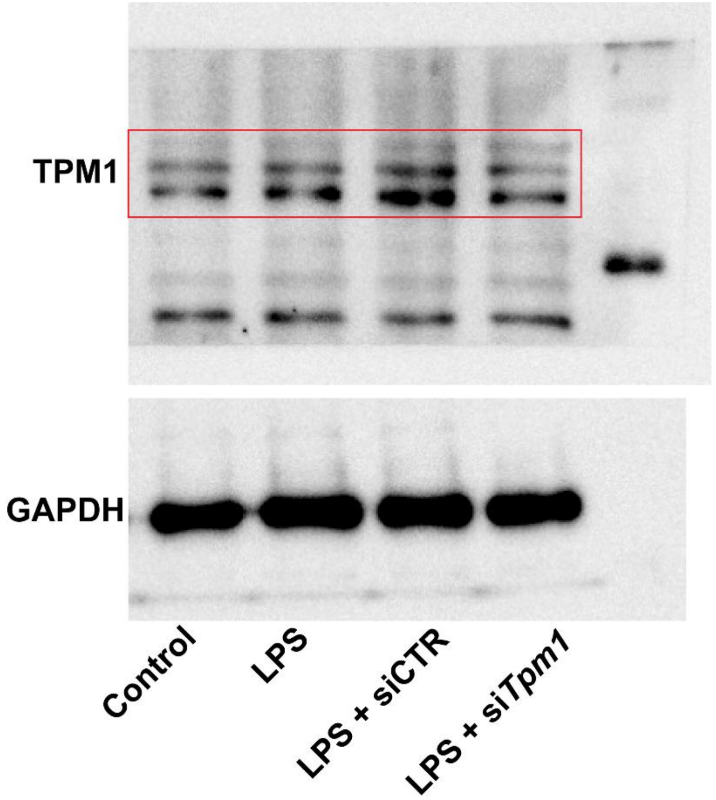

fig. S10 Full, uncropped gel images corresponding to Fig. S2 A–B. Western blots showing TPM1 expression in BV2 microglial cells treated with siTpm1 or siCTR followed by LPS stimulation. The boxes indicate the original TPM1 bands presented in **Fig. S2 A**.

Supplementary table 1
Table S1
Primer lists

Gene Name	Sequence (5'-3')
Tpm1 (Forward)	CTGAAGATGCTGACCGGAAGT
Tpm1 (Reverse)	TGGTTACTGATCTCTCTGCAAACTC
Tnfα (Forward)	ATGGCCTCCCTCTCATCAGT
Tnfα (Reverse)	TTTGCTACGACGTGGGCTAC
Il1β (Forward)	AATGCCACCTTTTGACAGTGATG
Il1β (Reverse)	GGAAGGTCCACGGGAAAGAC
Il6 (Forward)	GGGACTGATGCTGGTGACAA
Il6 (Reverse)	AGCATTGGAAATTGGGGTAGGA
Cox2 (Forward)	CTTCGGGAGCACAACAGAGT
Cox2 (Reverse)	AAGTGGTAACCGCTCAGGTG
Nos2 (Forward)	CCTCCTCCACCCTACCAAGT
Nos2 (Reverse)	CACCCAAAGTGCTTCAGTCA
Bax (Forward)	GAACCATCATGGGCTGGACA
Bax (Reverse)	AGCCACCCTGGTCTTGGAT
Caspase-3 (Forward)	GGAGCAGCTTTGTGTGTGTG
Caspase-3 (Reverse)	AGCCTCCACCGGTATCTTCT
Bcl2 (Forward)	AGTACCTGAACCGGCATCTG
Bcl2 (Reverse)	GGTATGCACCCAGAGTGATG
Gapdh (Foward)	GAAGGTCGGTGTGAACGGAT
Gapdh (Reverse)	TACTTGGCAGGTTTCTCCAGG
Ccnb1 (Forward)	AAGGCCAAGGTCAGTATGGC
Ccnb1 (Reverse)	TCGGGCTTGGAGAGGGATTA
Ccnb2 (Forward)	GCTAGCTCCCAAGGATCGTC
Ccnb2 (Reverse)	ATCCACCTCTCCGGCTTTTG
Ctsd (Forward)	TACTCCATGCAGTCATCGCC
Ctsd (Reverse)	CAAAGACCGGAAGCACGTTG
Apoe (Forward)	CCGACATGGAGGATCTACGC
Apoe (Reverse)	CTCCATCAGGTTTGCCCACT
P21 (Forward)	GCAAAGTGTGCCGTTGTCTC
P21 (Reverse)	CGTCTCCGTGACGAAGTCAA
Cdk1 (Forward)	AAGTGTGGCCAGAAGTCGAG
Cdk1 (Reverse)	TCGTCCAGGTTCTTGACGTG
Tubb4b (Forward)	GAGGTAATCAGCGACGAGCA
Tubb4b (Reverse)	CTTGGGGGAAGGTACCACAC
Tuba1c (Forward)	GTCCTGGACAGGATTCGCAA
Tuba1c (Reverse)	TCAGCACTATCTGCCCCAAC
Bnip3 (Forward)	AACAGCACTCTGTCTGAGGAA
Bnip3 (Reverse)	GGTCGACTTGACCAATCCCAT
Igf1 (Forward)	GGAAAATCAGCAGCCTTCCA
Igf1 (Reverse)	CGGGGACTTCTGAGTCTTGG

Itgav (Forward)	TGGTCGACTGGATAGAGGCA
Itgav (Reverse)	CTGAATGCCCCAGGTGATGT
Itgb1 (Forward)	GCTCCGGCCAGAAGACATTA
Itgb1 (Reverse)	AAGCCAATGCGGAAGTCTGA
Msr1 (Forward)	CGCACGTTCAATGACAGCAT
Msr1 (Reverse)	TGTCCTCCTGTTGCTTTGCT
Cd14 (Forward)	AAGGGTACAGCTGCAAGGAC
Cd14 (Reverse)	GGAGCAAAGCCAGAGTTCCT
Axl (Forward)	CCAGGTCCTCGTGCGTTG
Axl (Reverse)	TGGGGGTTCACTCACCAGAT
Jun (Forward)	TCGTTCCTCCAGTCCGAGAG
Jun (Reverse)	AGAAGGTCCGAGTTCTTGGC
Fos (Forward)	GGAGAATCCGAAGGGAACGG
Fos (Reverse)	GCAATCTCAGTCTGCAACGC
Fabp5 (Forward)	GGTCAAAACCGAGAGCACAG
Fabp5 (Reverse)	TGCCATCAGCTGTCGTTTCA
Apoc1 (Forward)	CCTGATTGTGGTCGTAGCCA
Apoc1 (Reverse)	CGGGCCTTGTCTTCCAAAGT
Plin2 (Forward)	GGCCAAACAAAAGAGCCAGG
Plin2 (Reverse)	CCTCAGACTGCTGGACCTTC
Lypla1 (Forward)	ACAGGCAGCAGAAACCGTAA
Lypla1 (Reverse)	AAACGAAGCCCGAAGTGGAA
Sp1 (Forward)	TCTTCAGGCCCTTCAAGCAG
Sp1 (Reverse)	AGGCAATGGGTGTTAGGGTG
Soat1 (Forward)	GGAGAATCCTGAGCAAGATGA
Soat1 (Reverse)	CATGGTAATGTGACCATTTCTGT
Cd45 (Forward)	TCAAAGTGACCCCTTACCTGC
Cd45 (Reverse)	CAAGGCTGGGGGTATCAACA
Lgals9 (Forward)	AAGGGGCGCAAACAGAAAAC
Lgals9 (Reverse)	TGGACTTGGACGGGTAAAGC
Mapkapk2 (Forward)	GGAGGAAGTGCCTGCTGATT
Mapkapk2 (Reverse)	CAGAGGGTTGGATGCGTCTT
Mapk6 (Forward)	GCATCTACTCCTTCCCGACG
Mapk6 (Reverse)	ACAGAGCTCTTGGGTCAAGC

Sequences for siRNAs

**Oligonucleotides Resource**	**Sequence (5'-3')**
mm-*Apoe*-si-1 (Sense)	GGAAGUAAAGGCUUACAAATT
mm-*Apoe*-si-1 (Anti-sense)	UUUGUAAGCCUUUACUUCCTT
mm-*Apoe*-si-2 (Sense)	GACUCGGGCAGUACCGCAATT
mm-*Apoe*-si-2 (Anti-sense)	UUGCGGUACUGCCCGAGUCTT
mm-*Apoe*-si-3 (Sense)	CCAGGAGAAUCAAUGAGUATT
mm-*Apoe*-si-3 (Anti-sense)	UACUCAUUGAUUCUCCUGGTT

mm-*Fabp5*-si-1 (Sense)	CGACUGUGUUCUCUUGUAACCTT
mm-*Fabp5*-si-1 (Anti-sense)	GGUUACAAGAGAACACAGUCGTT
mm-*Fabp5*-si-2 (Sense)	GGGAAGGAGAGCACGAUAACATT
mm-*Fabp5*-si-2 (Anti-sense)	UGUUAUCGUGCUCUCCUUCCCTT
mm-*Fabp5*-si-3 (Sense)	CAUGGACCUUCAUGUCAAACUTT
mm-*Fabp5*-si-3 (Anti-sense)	AGUUUGACAUGAAGGUCCAUGTT
mm-*Cd45*-si-1 (Sense)	GAUAAUCUCUACUAUUCAACUTT
mm-*Cd45*-si-1 (Anti-sense)	AGUUGAAUAGUAGAGAUUAUCTT
mm-*Cd45*-si-2 (Sense)	GGAACAAGUGCGCAGAAUACUTT
mm-*Cd45*-si-2 (Anti-sense)	AGUAUUCUGCGCACUUGUUCCTT
mm-*Cd45*-si-3 (Sense)	GGAACAUUACUGUGAAUUACATT
mm-*Cd45*-si-3 (Anti-sense)	UGUAAUUCACAGUAAUGUUCCTT
mm-*Lgals9*-si-1 (Sense)	GGAUAUCAACACUCUAGAAGUTT
mm-*Lgals9*-si-1 (Anti-sense)	ACUUCUAGAGUGUUGAUAUCCTT
mm-*Lgals9*-si-2 (Sense)	GGUGAUGGUGAACAAGAAAUUTT
mm-*Lgals9*-si-2 (Anti-sense)	AAUUUCUUGUUCACCAUCACCTT
mm-*Lgals9*-si-3 (Sense)	GAUGCUACGAGGUUCCAUAUCTT
mm-*Lgals9*-si-3 (Anti-sense)	GAUAUGGAACCUCGUAGCAUCTT
mm-*Mapk6*-si-1 (Sense)	CGAUGAAGAAGAAGUCCAAGUTT
mm-*Mapk6*-si-1 (Anti-sense)	ACUUGGACUUCUUCUUCAUCGTT
mm-*Mapk6*-si-2 (Sense)	GCAUCAGUCUGCUGACGUAGUTT
mm-*Mapk6*-si-2 (Anti-sense)	ACUACGUCAGCAGACUGAUGCTT
mm-*Mapk6*-si-3 (Sense)	GGAUCAUGGAUCCUCAUUAUUTT
mm-*Mapk6*-si-3 (Anti-sense)	AAUAAUGAGGAUCCAUGAUCCTT
